# Gene expression changes occurring at bolting time are associated with leaf senescence in Arabidopsis

**DOI:** 10.1002/pld3.279

**Published:** 2020-11-08

**Authors:** Will E. Hinckley, Judy A. Brusslan

**Affiliations:** ^1^ Department of Biology New York University New York NY USA; ^2^ Department of Biological Sciences California State University Long Beach, Long Beach CA USA

**Keywords:** Arabidopsis trithorax, bolting, flowering time, gene regulatory networks, leaf senescence, transcriptomics

## Abstract

In plants, the vegetative to reproductive phase transition (termed bolting in Arabidopsis) generally precedes age‐dependent leaf senescence (LS). Many studies describe a temporal link between bolting time and LS, as plants that bolt early, senesce early, and plants that bolt late, senesce late. The molecular mechanisms underlying this relationship are unknown and are potentially agriculturally important, as they may allow for the development of crops that can overcome early LS caused by stress‐related early‐phase transition. We hypothesized that leaf gene expression changes occurring in synchrony with bolting were regulating LS. *ARABIDOPSIS TRITHORAX (ATX)* enzymes are general methyltransferases that regulate the adult vegetative to reproductive phase transition. We generated an *atx1, atx3, and atx4* (*atx1,3,4*) triple T‐DNA insertion mutant that displays both early bolting and early LS. This mutant was used in an RNA‐seq time‐series experiment to identify gene expression changes in rosette leaves that are likely associated with bolting. By comparing the early bolting mutant to vegetative WT plants of the same age, we were able to generate a list of differentially expressed genes (DEGs) that change expression with bolting as the plants age. We trimmed the list by intersection with publicly available WT datasets, which removed genes from our DEG list that were *atx1,3,4* specific. The resulting 398 bolting‐associated genes (BAGs) are differentially expressed in a mature rosette leaf at bolting. The BAG list contains many well‐characterized LS regulators (*ORE1, WRKY45, NAP, WRKY28*), and GO analysis revealed enrichment for LS and LS‐related processes. These bolting‐associated LS regulators may contribute to the temporal coupling of bolting time to LS.

## 1 INTRODUCTION

Leaf senescence (LS) is the sequential death of older leaves, one‐by‐one, as the plant matures, while whole plant senescence is the simultaneous death of all leaves at the end of the growing season in monocarpic species (Nooden et al., 1997). A visual hallmark of LS is leaf yellowing, caused by chlorophyll degradation (Ougham et al., 2008; Tamary et al., 2019). During these processes, nitrogen (most commonly in the forms of nitrate, asparagine, and glutamine) and other macromolecules are recycled from dying leaves (sources) and relocated to growing tissues (sinks), including the reproductive organs (Havé et al., 2017). A better understanding of the regulation of LS may have important agricultural implications on yield and nutrition content.

Endogenous signaling molecules that control LS have been well characterized. Ethylene, abscisic acid (ABA), jasmonic acid (JA), salicylic acid (SA), and reactive oxygen species (ROS) are known to promote both age‐dependent and dark‐induced LS (Jing et al., 2005; Khanna‐Chopra, 2012; Lim et al., 2007; Yuehui et al., 2002; Zhang et al., 2013, 2020; Zhao et al., 2017). Many genetic regulators of LS have also been identified (Ay et al., 2014; Brusslan et al., 2015; Chen, Lu, et al., 2016; Hinckley et al., 2019; Keqiang et al., 2008; Kim, Park, et al., 2018; Liu et al., 2019; Wang et al., 2019; Woo et al., 2013; Woo et al., 2019; Zheng et al., 2020). There are multiple large TF families that are commonly associated with age‐dependent and dark‐induced LS (*WRKY, NAC, ERF*) (Bakshi & Oelmüller, 2014; Jiang et al., 2017; Kim et al., 2016; Koyama, 2014; Koyama et al., 2013; Li, Li, et al., 2018). Many individual TFs have also been shown to regulate LS; for example, *NAP, WRKY53*, *WRKY75,* and *ORE1* are positive regulators and *JUB1, WRKY54,* and *WRKY70* negatively regulate LS (Guo et al., 2017; Lei et al., 2020; Miao et al., 2004; Qiu et al., 2015; Zentgraf & Doll, 2019). Furthermore, stress, defense, and LS signaling overlap and some TFs are known to bridge stress and LS signaling (*SAG113, NAP, WRKY53*) (Asad et al., 2019; Kim et al., 2013; Sade et al., 2018; Yang et al., 2014).

While many regulators have been identified that function at the onset of or during LS, less is known about developmentally early regulators of LS. Kim et al. uncovered the NAC troika, consisting of three Arabidopsis NAC TFs that act in young rosette leaves to prevent early LS (Kim, Park, et al., 2018). To our knowledge, this represents the earliest known regulation of LS in Arabidopsis.

Generally, preceding LS is the vegetative to reproductive phase transition, which in Arabidopsis is termed bolting or flowering: the development of the primary inflorescence that produces cauline leaves, inflorescence meristems, and floral meristems. Many different environmental and autonomous cues can induce flowering independently; however, all pathways converge on conserved master flowering time regulators: *FT and SOC1* (Mouradov et al., 2002; Song et al., 2018). Stress can also induce flowering (Takeno, 2016; Wada & Takeno, 2010). The function of stress signaling in both flowering time and LS may serve as a link that allows reproduction during stress.

Many studies in Arabidopsis show a relationship between bolting time and leaf and/or whole plant senescence. While one study noted a negative correlation between flowering time and LS (Luquez et al., 2006), many other studies using more conventional methods describe a positive correlation between flowering time and LS. (Balazadeh et al., 2008; Huang et al., 2019; Jiang et al., 2019; Kim, Park, et al., 2018; Li, Zhang, et al., 2018; Upadhyay et al., 2014; Yan et al., 2017). For example, *KHZ1* and *KHZ2* encode redundant KH domain Zn‐finger TFs, and double *khz1khz2* mutants bolt late and show delayed LS and whole plant senescence. Overexpression of *KHZ1* or *KHZ2* resulted in early bolting, LS, and whole plant senescence (Yan et al., 2017). As most recent studies support a positive correlation between bolting and LS, we hypothesized that there are LS‐related gene expression changes occurring in the rosette at bolting time. These gene expression changes could be contributing to the positive correlation between bolting time and LS.

We generated an *atx1 atx3 atx4* triple T‐DNA insertion mutant that displayed early bolting and early LS. We then used this mutant to model the temporal relationship between bolting and LS in an RNA‐seq time‐series experiment that compared the early bolting mutants to vegetative WT plants of the same age.

Our approach allowed the identification of leaf gene expression changes likely associated with bolting time. This list was then trimmed by intersection with developmentally similar publicly available WT datasets. The resulting list of 398 Bolting Associate Genes (BAGs) was enriched for LS and LS‐related GO terms, and includes many well‐characterized LS regulators. We found that 202 of these BAGs are included in the LS database (Li et al., 2020). We then produced a gene regulatory network (GRN) summarizing BAG interactions using machine learning (GENIE3) and trimmed it with publicly available DAP‐seq TF binding site data (Huynh‐Thu et al., 2010; O’Malley et al., 2016). This study shows that there are gene expression changes localized to the leaf and concomitant to bolting that may regulate LS in *Arabidopsis thaliana*.

## 2 MATERIALS AND METHODS

### 2.1 Plant growth conditions

*Arabidopsis thaliana* plants were grown in Sunshine^®^ Mix #1 Fafard^®^‐1P RSi soil (Sungro Horticulture), which was treated with Gnatrol WDG (Valent Professional Products) (0.3 g/500 ml H_2_O) to inhibit the growth of fungus gnat larvae. Plants were subirrigated with Gro‐Power 4‐8‐2 (Gro‐Power, Inc.) (8 ml per gallon), and grown in Percival AR66L2X growth chambers under a 20:4 light:dark diurnal cycle (Long Day) with a light intensity of 28 μmoles photons m^−2^ s^−1^. The low light intensity prevents light stress in older leaves, which was evident as anthocyanin accumulation at higher light intensities. To compensate for the reduced light intensity, the day length was extended. The petiole of the sixth leaf to emerge was marked with a thread on individual plants.

### 2.2 Genotype analysis

The *atx1 atx3 atx4* triple mutant was generated by crossing two double mutants (*atx1 atx3*, and *atx3 atx4*). Alleles and corresponding primers can be found in Data File S1, Sheet 6. Genomic DNA was isolated from two–three leaves using Plant DNAzol Reagent (Thermo Fisher) following manufacturer's instructions. Pellets were dried at room temperature for at least 2 hr, and resuspended in 30 μl TE (10 mM Tris, pH 8.0, 1 mM EDTA) overnight at 4°C. One microliter of genomic DNA was used as a template in PCR reactions with primers listed in Data File S1, Sheet 6. All standard PCR reactions were performed with a 57°C annealing temperature using *Taq* polymerase with Standard *Taq* Buffer (New England Biolabs).

### 2.3 Chlorophyll analysis

One hole punch was removed from each marked leaf and incubated in 800 µl N,N‐dimethyl formamide (DMF) overnight in the dark. A total volume of 200 µl of sample was transferred to a quartz microplate (Molecular Devices) and absorbance at 664 and 647 nm was measured with a BioTek Synergy H1 plate reader. Absorbance readings were used to determine chlorophyll concentration (Porra et al., 1989). For each genotype/condition, *n* = 6 single‐hole punches from six individual plants.

### 2.4 Gene expression

Total RNA was isolated from leaf 6 using Trizol reagent. Extracted RNA of 1,000 ng was used as a template for cDNA synthesis using MMLV reverse transcriptase (New England Biolabs) and random hexamers to prime cDNA synthesis. The cDNA was diluted 16‐fold and used as a template for real‐time qPCR using either ABsolute QPCR Mix, SYBR Green, ROX (Thermo Scientific) or qPCRBIO SyGreen Blue Mix Hi‐Rox (PCR Biosystems), in Step One Plus or Quant Studio 6 Flex (Thermo Fisher) qPCR machines. All real‐time qPCR reactions were run with a 61°C annealing temperature, and normalized to *ACT2*.

### 2.5 RNA‐seq library construction

Ten plants per line at each time point were selected for harvesting from the developmentally synchronized group of bolting *atx1,3,4* TM or WT control plants. All harvesting was completed between 8:00 and 10:00 a.m. to prevent interference by diurnal gene expression changes. Leaf 6 was harvested from all 10 plants and the 10 leaves were immediately flash‐frozen together. A mortar and pestle were used to grind tissue in liquid nitrogen. Homogenized tissue was separated evenly into three tubes to be treated as three replicates. The Breath‐Adaptive Directional sequencing (BrAD‐seq) (Townsley et al., 2015) protocol was completed to generate cDNA libraries. A 1/50 dilution of the final library was used with *ACT2* primers in qPCR to check for library amplification consistency. The Illumina‐ready cDNA libraries were sequenced at the UC Irvine Genome High‐Throughput Facility (GHTF).

### 2.6 RNA‐seq data analysis

Two types of data were used for differential expression analysis. Rsubread was used to align reads to the TAIR10 genome. Noiseq normalized aligned counts by read length and library size to generate an FPKM dataset. HTS filter filtered out low reads. This FPKM dataset was used for the *T* test analysis. To prepare for the DeSeq2 and edgeR analyses, raw data were aligned to the TAIR10 genome and counted using Kallisto. Data were then exported to R and rounded to the nearest integer for differential expression analyses. DESeq2 was completed using a 2 factor (Genotype + Time + Genotype:Time) model that treated time as a continuous variable, rather than a category (Data File S3). An 0.05 p‐value and adjusted P‐Value (FDR) cutoffs were used to determine significance. PCA was completed with DESeq2 (Love et al., 2014). Comparisons were made at each time point with edgeR with a p‐value cutoff of 0.05 and a 1.5‐fold change cutoff for significance (Data File S3) (Robinson et al., 2009). A simple *T* test–based approach was written in R that used a 0.05 p‐value and 2‐fold change cutoff (Data File S3) and used to compare WT and *atx1,3,4* TMs at each time point. For all three approaches, WT was compared with TM1 and TM2 separately. The overlap between the TM1 versus WT and TM2 versus WT DEG lists was determined for each statistical method separately using R. The intersection of these DEG lists between the three methods was then identified using http://www.interactivenn.net/. Heatmaps were generated using heatmap.2.

### 2.7 GRN construction

FPKM data for the 398 BAGs were used as input into GENIE3 (Huynh‐Thu et al., 2010). BAG TFs were assigned as regulators. The resulting set of interactions was uploaded to ConnecTF.org for a precision recall analysis to trim the network with DAP‐seq binding data. The precision recall analysis indicated the machine learning performed better than if the interactions were assigned at random (Figure S2b). The trimmed network was annotated in R and uploaded to Cytoscape for visualization.

## 3 RESULTS

### 3.1 *atx1,3,4* Triple‐mutant phenotype

**FIGURE 1:**
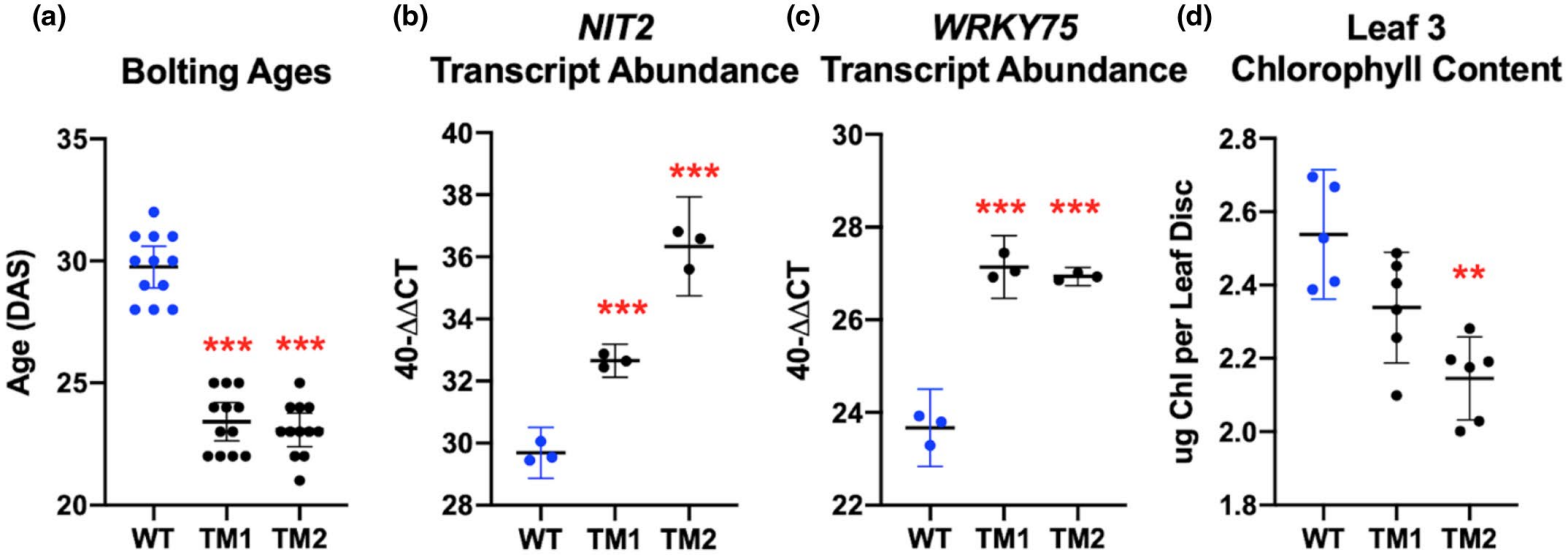
*atx1 atx3 atx4* triple‐mutant (TM) phenotypes. (a) A plant was considered to have bolted when the inflorescence extended 1 centimeter from the base of the rosette. (b and c) Real‐Time qPCR was used to measure the transcript abundance of two genetic LS markers, *NIT2* and *WRKY75,* in RNA isolated from leaf 6 at day 33. Individual data points shown are the averages of three technical replicates, generated from 6 plants. (d) A leaf disc obtained by hole‐punching leaf 3 was harvested at 33 days of age. For statistics, one‐way ANOVAs were run. Then, *T* tests with Bonferroni‐corrected *p*‐values were completed to determine significance. For all data, one representative replicate of 3 is shown. Results were similar in all three replicates, which can be found in Data File S1_Sheets 1–4. Error bars display 95% confidence intervals.

Class III Arabidopsis trithorax (ATX) histone methyltransferases methylate H3K4 (Pontvianne et al., 2010). Different ATX enzymes catalyze different methyltransferase activities (mono‐, di‐, tri‐methylation), and some *atx* single mutants display more severely altered developmental phenotypes than others (Chen, Luo, et al., 2017; Tamada et al., 2009; Yun et al., 2012). While double‐mutant combinations displayed early flowering, our single *atx1*, *atx3*, and *atx4* mutants and double‐mutant combinations did not display a detectable change in LS, prompting the isolation of a homozygous *atx1 atx3 atx4* triple mutant (*atx1,3,4*). We isolated two *atx1,3,4* triple mutants (TM1 and TM2), which contain the same alleles but are derived from different F_1_ plants from the same cross, and are independent isolates of the same genotype. The *atx1,3,4* triple‐mutant genotype was confirmed both by PCR and RT‐PCR (Figure S1). The *atx1,3,4* mutants displayed significantly early bolting (Figure 1a) and significantly early LS, quantified by *NIT2* and *WRKY75* mRNA induction (Figure 1b,c) and by chlorophyll loss (Figure 1d). We also confirmed that early bolting translated to an early flower formation phenotype (Data File S1, Sheet_5), demonstrating bolting is an appropriate phenotype marker for the vegetative‐reproductive transition.

The accumulation of H3K4me3 downstream of the *FLOWERING LOCUS C (FLC)* TSS is associated with high expression of *FLC*, a flowering inhibitor, thereby preventing the vegetative to reproductive transition (Pien et al., 2008; Yun et al., 2012). The early bolting phenotype in the *atx1,3,4* TMs is likely due to decrease in H3K4me3 accumulation at the *FLC* locus and decreased *FLC* gene expression, which has been observed in other *atx* mutants (Pien et al., 2008).

K4‐SURGs are genes that gain the H3K4me3 mark at the same time their gene expression increases during LS (Brusslan et al., 2015). *NIT2* encodes a nitrilase and is a robust K4‐SURG that serves as an mRNA marker for LS (Brusslan et al., 2012). *WRKY75* is a well‐characterized positive regulator of LS (Guo et al.,^2017^) that was also identified as a K4‐SURG. H3K4me3 is an activating mark; thus, if hypomethylation caused by *atx1,3,4* mutation affected *NIT2* or *WRKY75,* we would expect lower gene expression, but we detected the opposite (Figure 1b,c). Other than early bolting and LS, there were no other apparent phenotype changes in the *atx1,3,4* TMs compared to WT. This led us to hypothesize that *NIT2* and *WRKY75* induction were not directly caused by H3K4me3 changes, rather the coupling of LS to bolting time might be responsible. The genetic mechanism behind this temporal relationship has not been defined, and it became our goal to identify leaf‐localized gene expression changes associated with the bolting event that may be regulating LS.

### 3.2 RNA‐seq time‐series experimental design

**FIGURE 2:**
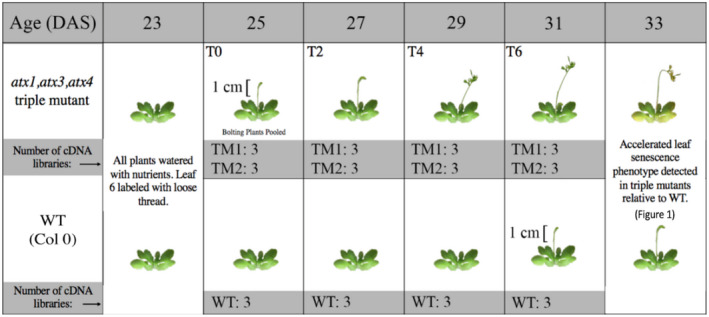
RNA‐seq time‐series experimental design. WT, *atx1,3,4* Triple‐Mutant 1 (TM1), and *atx1,3,4* Triple‐Mutant 2 (TM2) were grown in long‐day conditions (*n* = 54 per genotype). Bolting age was scored and mutant plants recorded at the peak of TM bolting (days 24 and 25) were grouped. Ten bolting mutant plants were randomly selected from this group four times in two‐day increments to ensure that plants were developmentally similar. Ten WT control plants were also harvested at each time point. Leaf 6 was harvested and stored at −80°C. Leaves were homogenized in liquid nitrogen with a mortar and pestle and then separated into three tubes, which were treated as three replicates/libraries.

We completed an RNA‐seq time‐series experiment that compared the early bolting *atx1,3,4* TMs to vegetative WT Col‐0 plants of the same age over a 6‐day time course (Figure 2). It was important to include a bolting line and a vegetative control of the same age to allow for the differentiation of DEGs associated with bolting from DEGs associated with age. Using early bolting mutants was advantageous as it engendered more synchronous bolting and prevented a more prolonged age bias that could be introduced by using late‐bolting mutants. If LS‐related signaling was being initiated in leaves by bolting, we would expect to see LS‐related differentially expressed genes (DEGs) between the *atx1,3,4* TMs and WT, and enrichment of LS‐related biological processes within these DEGs.

Mutant plants that bolted at the peak of *atx1,3,4* TM bolting (Day 24–25, T0) were grouped into a cohort. We randomly selected individuals from this cohort for leaf 6 harvesting, which began at T0 and continued in 2‐day increments (T2, T4, and T6). This synchronization to bolting ensured the *atx1,3,4* plants were developmentally similar. WT leaf 6 control tissue was harvested at the same time points. At T6, one WT plant had bolted, but all other WT plants were vegetative. This design allowed us to differentiate gene expression changes associated with bolting versus those associated with age. cDNA libraries were prepared and subject to high‐throughput sequencing (Data File S2 reports FPKM values).

**FIGURE 3:**
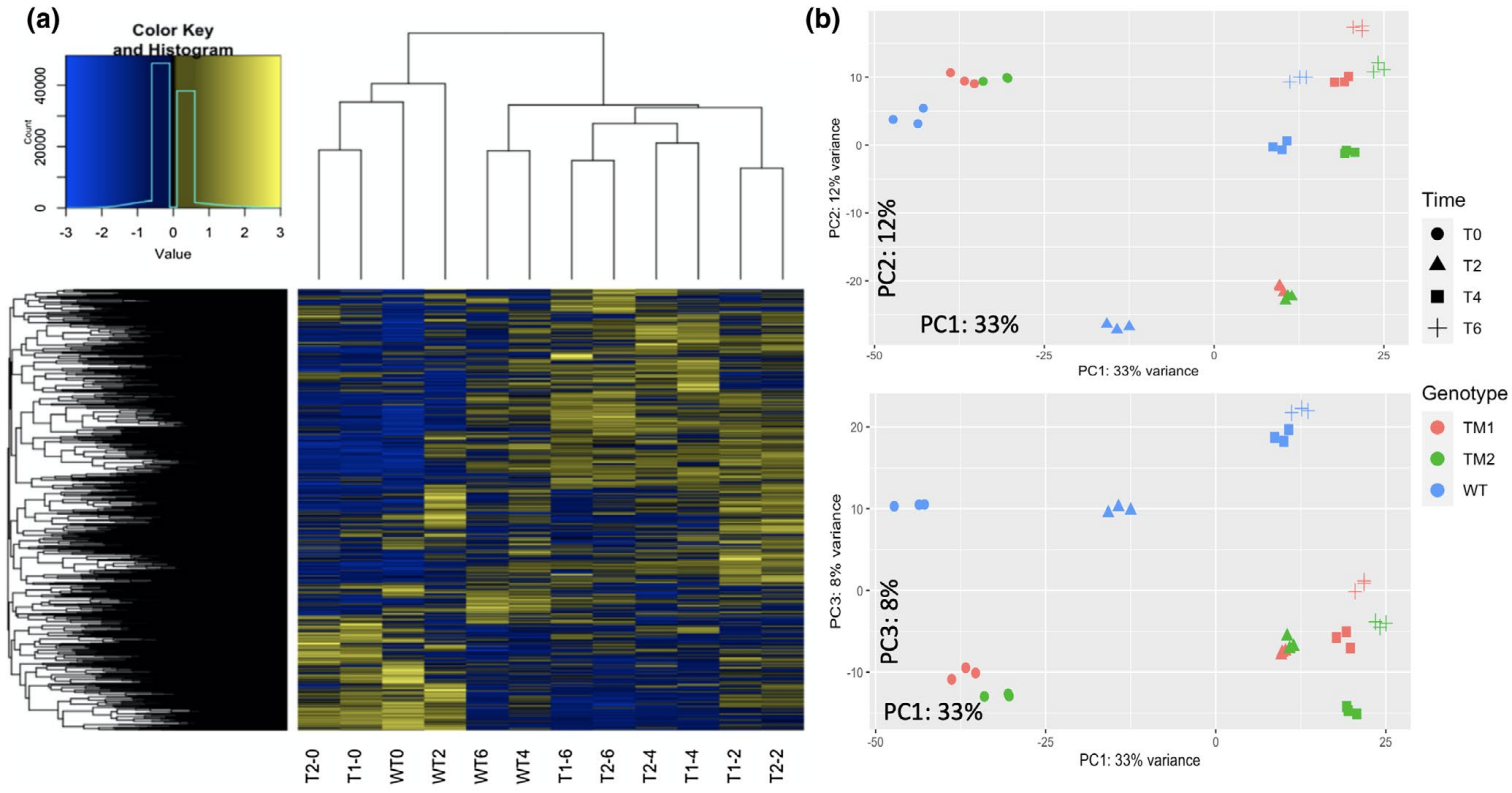
Transcriptome comparisons. Hierarchical clustering was used to generate a heatmap of transcriptomes, which represent mean expression levels of the three replicates per line at each time point. (T1 = *atx1,3,4* TM1, T2 = *atx1,3,4* TM2). PCA was completed using the DeSeq2 package, and results for the first three principal components are shown. (Data File S3 contains the code used to generate the PCA plots).

As expected, hierarchical transcriptome clustering showed TM1 and TM2 are similar at each time point (Figure 3a). All T0 samples clustered together, regardless of genotype, while the T2, T4, and T6 transcriptomes from bolting *atx1,3,4* TMs cluster away from the vegetative WT samples (T0 and T2). The clustering of all T0 samples is likely because the mutants had just begun the phase transition. As they progress further into the reproductive phase, they cluster away from WT. WT samples from T4 and T6 cluster with the *atx1,3,4* TM bolting samples, likely because they are nearing the vegetative‐reproductive transition. At T6, one WT plant had bolted. Similar clustering patterns were also seen using Principal Component Analysis (PCA, Figure 3b). PCA shows how samples separate both by time and genotype/bolting phenotype. PCA also highlights that age affects our samples, as samples separate sequentially by harvesting age (Figure 3b). Extended PCA analysis shows how samples separate throughout the first five PCs (Figure S2a).

### 3.3 RNA‐seq data analysis strategy

**FIGURE 4:**
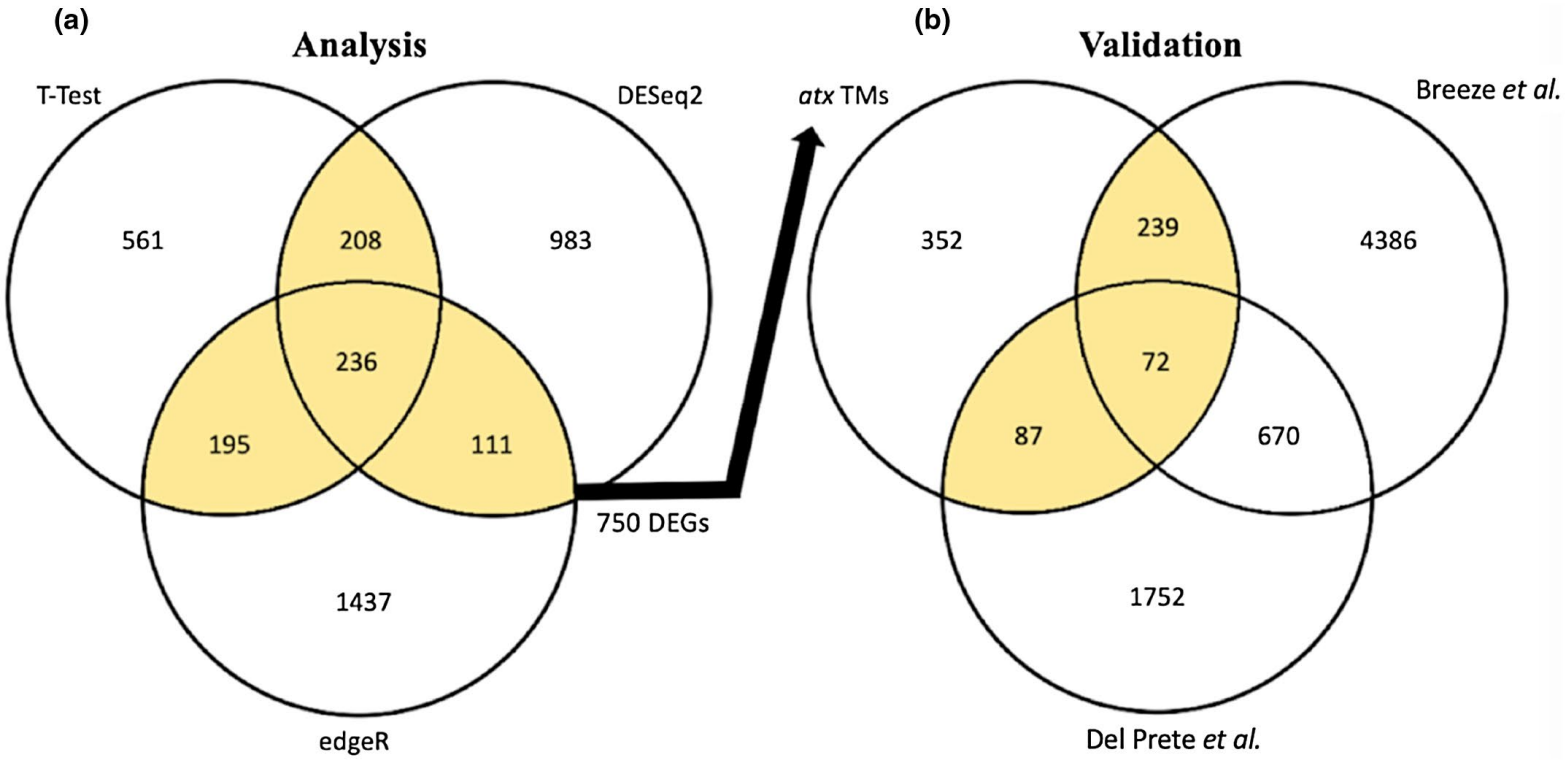
Differential expression analysis and validation. (a) DEGs lists from our *T* test method (1200), DESeq2 (1536), and edgeR (1978) were overlapped. DEGs identified by at least two of the three statistical methods (750) were considered for validation. (b) In order to be considered bolting associated, a gene needed to be differentially expressed in our *atx1,3,4* triple‐mutant analysis and at least one of the publicly available WT time‐series experiments. Highlighted portions in the Venn diagram mark those selected and used for validation from our analysis, or which genes were considered to be BAGs after validation. Data File S4 contains gene lists.

Three methods were used to determine differential expression (Figure 4). A gene needed to be identified by at least two different statistical methods to be considered a DEG. A (Genotype + Time + Genotype:Time) design was used to run an LRT test with (~Time) as a reduced model in DESeq2, which treated time as a continuous variable and removed genes that show the same expression pattern over time in all samples (Love et al., 2014). edgeR was used to compare each mutant to WT separately at each time point, meaning time was treated as a factor (Robinson et al., 2009). Lastly, a *T* test–based method was written in R (All code used for differential expression analysis can be found in Data File S3). The use of multiple methods increased stringency to prevent spurious detection of differential gene expression. Our list of 750 DEGs (Data File S4, Sheet 3) contained many bolting/flowering time regulators (*SOC1, FT, FLC, MED18, FPA, CIB1, CIB5, SEP1, SEP3, MAF4, and MAF5*), providing confidence that our harvesting method and differential expression analysis isolated bolting‐related gene expression changes.

We then sought to validate these DEGs in other datasets that studied WT Col‐0 plants undergoing the vegetative‐reproductive phase transition (Figure 4, Data File S4, Sheet 4). While not strictly synchronized to bolting, Breeze *et al*. completed a similar developmental time‐series experiment in Arabidopsis (Breeze et al., 2011). Leaf 7 was harvested from WT Col‐0 in 2‐day increments. We selected the time point at which plants began to flower in their time series (Day 21) and included the three subsequent time points. DEGs from these four time points were overlapped with our DEG list. Del Prete *et al*. identified genes downstream of *FT* and *SOC1* by monitoring transcriptome changes in WT Col‐0 Arabidopsis plants as they transitioned from short‐day (*SD*) to long‐day (LD) photoperiods (Del Prete et al., 2019). This photoperiod transition induces flowering, and while their transcriptomes were generated from pre‐bolting plants, the genetic changes associated with the *SD*‐LD transition are related to the phase transition.

Compared to our experiment, plants in these two experiments were solely WT genotypes, were not developmentally synchronized, were grown in different chambers, and DEG lists were generated using different statistical methods. However, all datasets covered the vegetative‐reproductive transition. It is important to note that *FT* and *SOC1* were both found to be differentially expressed in all three experiments, indicating canonical flowering time‐related gene expression was occurring in each dataset.

### 3.4 Bolting‐associated genes (BAGs)

In order to be considered a bolting‐associated gene (BAG), a gene had to be identified by at least two of the three statistical methods used in our analysis (Figure 4a), and it had to be validated in at least one of the WT time‐series experiments (Figure 4b). Default parameters on Genesect from VirtualPlant1.3 were used to determine that all gene lists displayed significant overlap (*p* < .001) (Katari et al., 2010). DEGs identified in our experiment that did not overlap with the WT experiments may be false positives or may be specifically associated with early flowering or *atx1,3,4* mutations. This approach retained genes that changed expression during bolting in WT plants and stringently identified 398 genes that are differentially expressed at bolting time (Highlighted portion of Figure 4b, Data File S4, Sheet 5).

**FIGURE 5:**
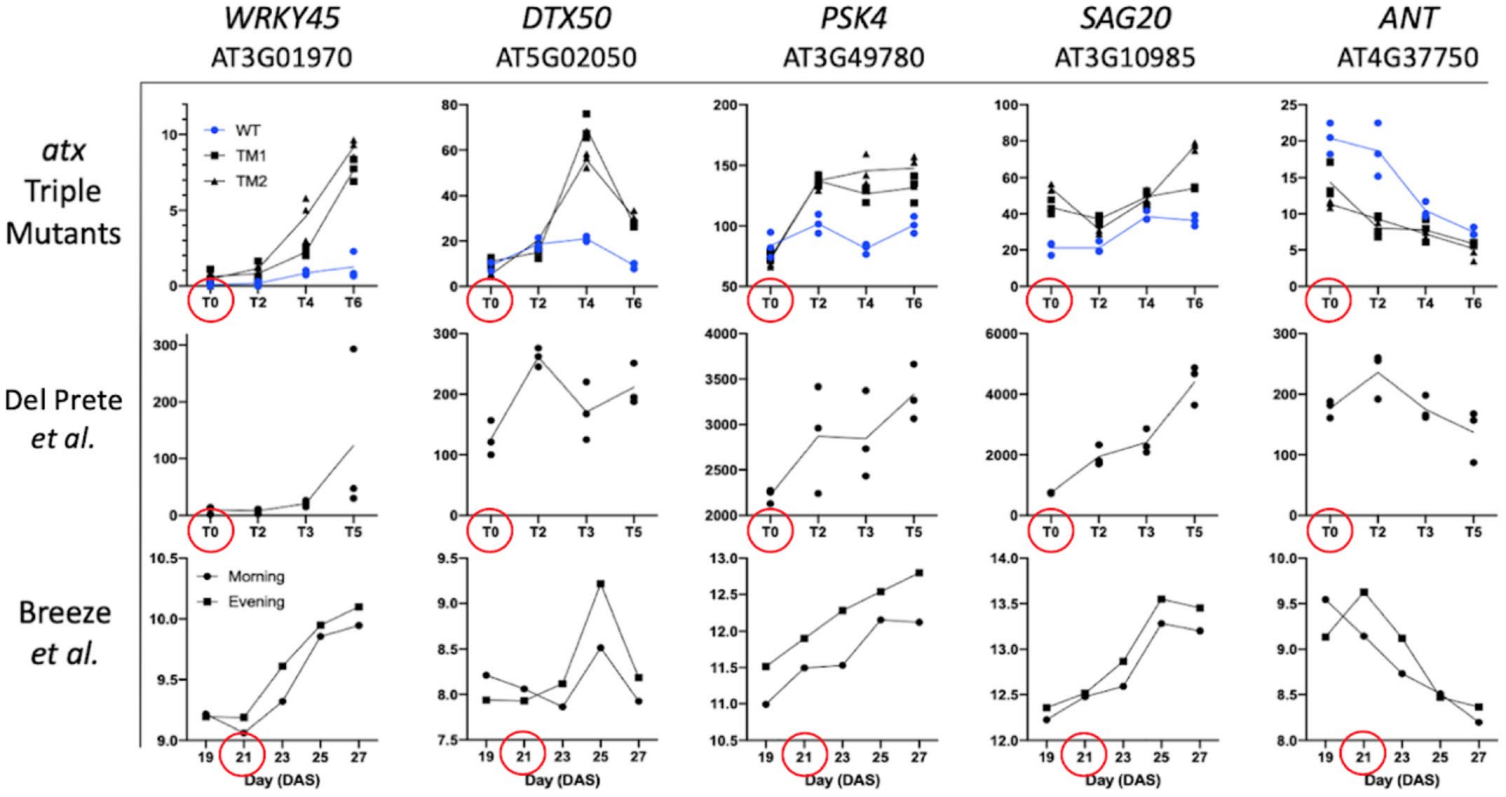
Examples of BAG expression across experiments. Raw mean data from each experiment were used to generate graphs in GraphPad PRISM. Data from the *atx1,3,4* TM and Del Prete *et al*. experiments represent FPKM from RNA‐seq, while Breeze et al. is Lowess normalized averaged (4 reps) signals in log space from a microarray experiment. Red circles indicate the time of phase transition in each experiment (bolting/flowering time for the *atx* triple mutants and Breeze et al., and time of photoperiod transition for Del Prete et al). Samples from plants undergoing the vegetative‐reproductive transition are shown in black while vegetative control plants are shown in blue.

A script was written in R to visualize the expression profiles of all 398 BAGs (Data File S5). Select gene expression profiles are shown along with the corresponding profiles from the WT time‐series experiments (Figure 5). *WRKY45* increased over time in all samples except for the vegetative WT control in our experiment. *DTX50* also showed strong induction in each experiment, although it decreased back to basal levels after 4 days in all datasets. *PSK4* increases at T0 and then maintains expression levels higher than WT at all subsequent time points, which corresponds to the clear induction of *PSK4* in both WT datasets. *SAG20* did not show as clear of a trend of expression over time, but it was consistently higher than vegetative WT levels. *SAG20* increases expression in both WT time series. *ANT* appears to decrease expression in all datasets undergoing the vegetative reproductive transition. WT follows the same trend, with a delayed decrease in *ANT* expression compared to the *atx1,3,4* TMs, likely as WT was nearing bolting time.

**FIGURE 6:**
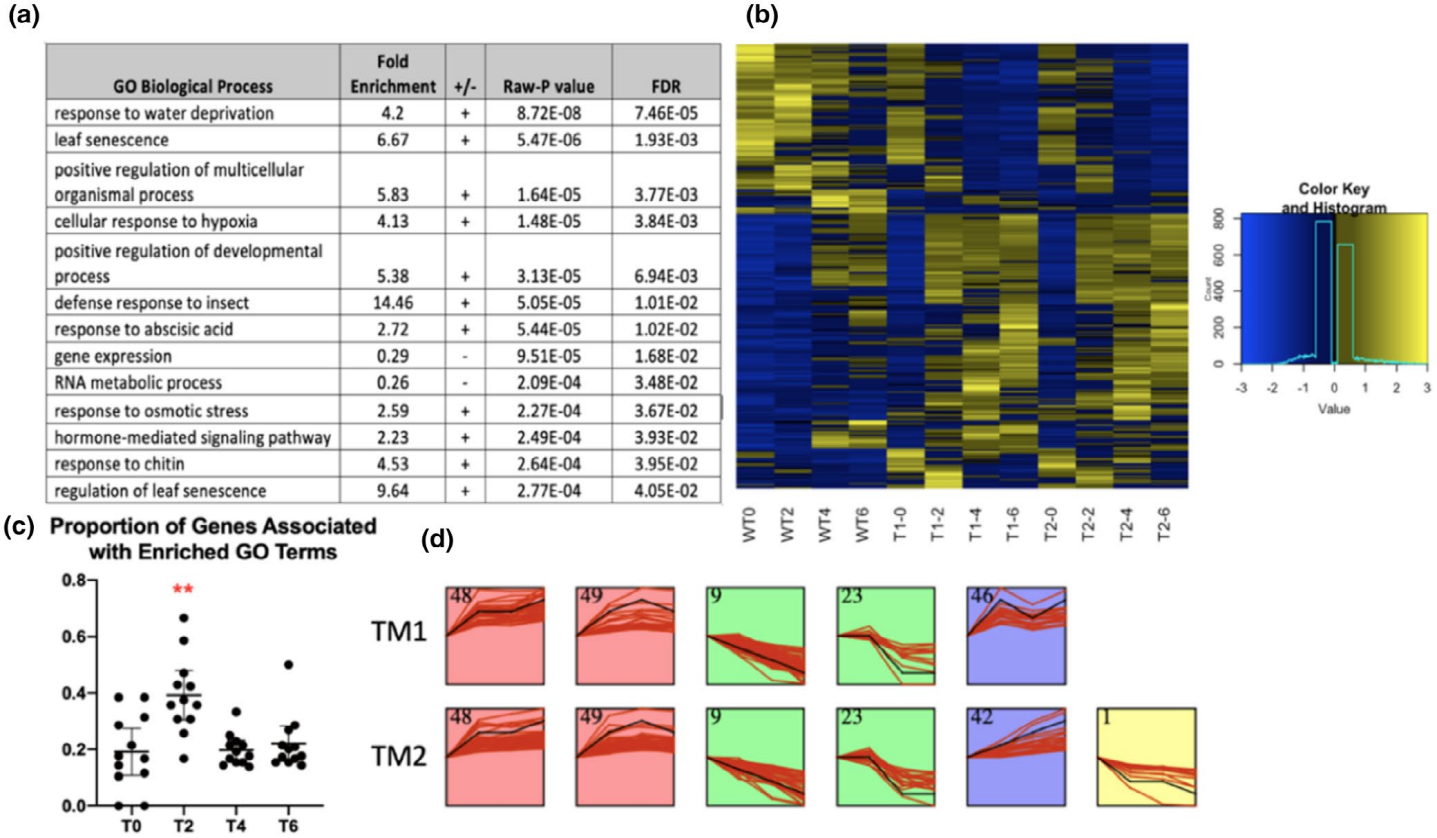
Bolting‐associated genes (BAGs). (a) Enriched GO terms from a Panther Gene Ontology analysis are shown. (b) A heatmap showing BAG expression levels in all 12 transcriptomes. (c) An ANOVA run on the time‐intersected enriched BAGs generated a significant P‐value (1.3E‐05). Pairwise *T* tests were then completed using a Bonferonni‐corrected p‐value (0.05/6 = 0.0083), which indicated that T2 was significantly different than all other time points [*p* = .0003 (T0), 0.0002 (T4), 0.0008 (T6)]. No other significant results were detected. Error bars show the 95 percent confidence intervals. (d) Short Time‐Series Expression Miner (STEM) was used to find significant clusters of BAG gene expression changes in TM1 and TM2.

The 398 BAGs are enriched for both LS and LS‐related biological processes (Figure 6a). The heatmap shows that the most dramatic differences in BAG expression between the *atx1,3,4* TMs and WT are seen between T0 and T2 (Figure 6b). We also confirmed that the subset of BAGs associated with enriched GO terms behaved similarly to the overall BAG list. The Panther Gene Ontology analysis allows extraction of the input DEGs associated with each enriched term (Mi et al., 2013; Thomas et al., 2003) (Data File S6). We intersected these input BAGs associated with individual‐enriched GO terms with the times that they were differentially expressed in our time‐series experiment (in TM2 from the edgeR analysis). As expected, among the genes associated with enriched GO terms, there was a significantly higher proportion of BAGs differentially expressed at T2 compared to all other time points (Figure 6c). BAGs changing expression more frequently at the time point most closely following the emergence of the bolt supports the hypothesis that the bolting event/phase transition is the stimulus of LS‐related signaling. We also note that at T4 and T6, BAG expression in WT plants becomes more similar to that of bolting TM plants, likely because these WT samples were nearing the vegetative‐reproductive transition. Short Time‐series Expression Miner (STEM) clustering analysis was completed to determine if TM1 and TM2 displayed consistent BAG gene expression patterns. STEM identified mostly the same significant gene expression clusters in both mutant lines (Figure 6d) (Ernst & Bar‐Joseph, 2006).

### 3.5 Potential novel early regulators of LS

We then wanted to identify genes within the BAG list that either regulate or are associated with LS. Li *et al*. generated a database of genes known to be associated with LS (LSD 3.0) (Li et al., 2020). Using VirtualPlant, we found a significant overlap between BAGs and the LSD 3.0 (*p* < .001). A total of 202 of the 398 BAGs (50.7%) were shared (Data File S4, Sheet 6). While some of these known LS‐associated BAGs might contribute to the coupling of LS to flowering time, we also wanted to find potential novel LS regulators. Kim et al completed a dark‐induced detached leaf senescence (DILS) RNA‐seq time‐series experiment with WT Col‐0 plants that we used to find genes that change expression during DILS (GSE99754) (Kim, Park, et al., 2018). We compared gene expression from T0 (before dark treatment) and T3 (3 days into dark treatment) using DESeq2 with default parameters and standard cutoffs (*p* < .05, FDR < 0.05, and a log2‐fold change >2) (Data File S4, Sheet 7). T3 was chosen because gene expression changes at later time points in DILS are shared among different triggers of LS (age dependent versus. dark induced) (Guo & Gan, 2012).

**FIGURE 7:**
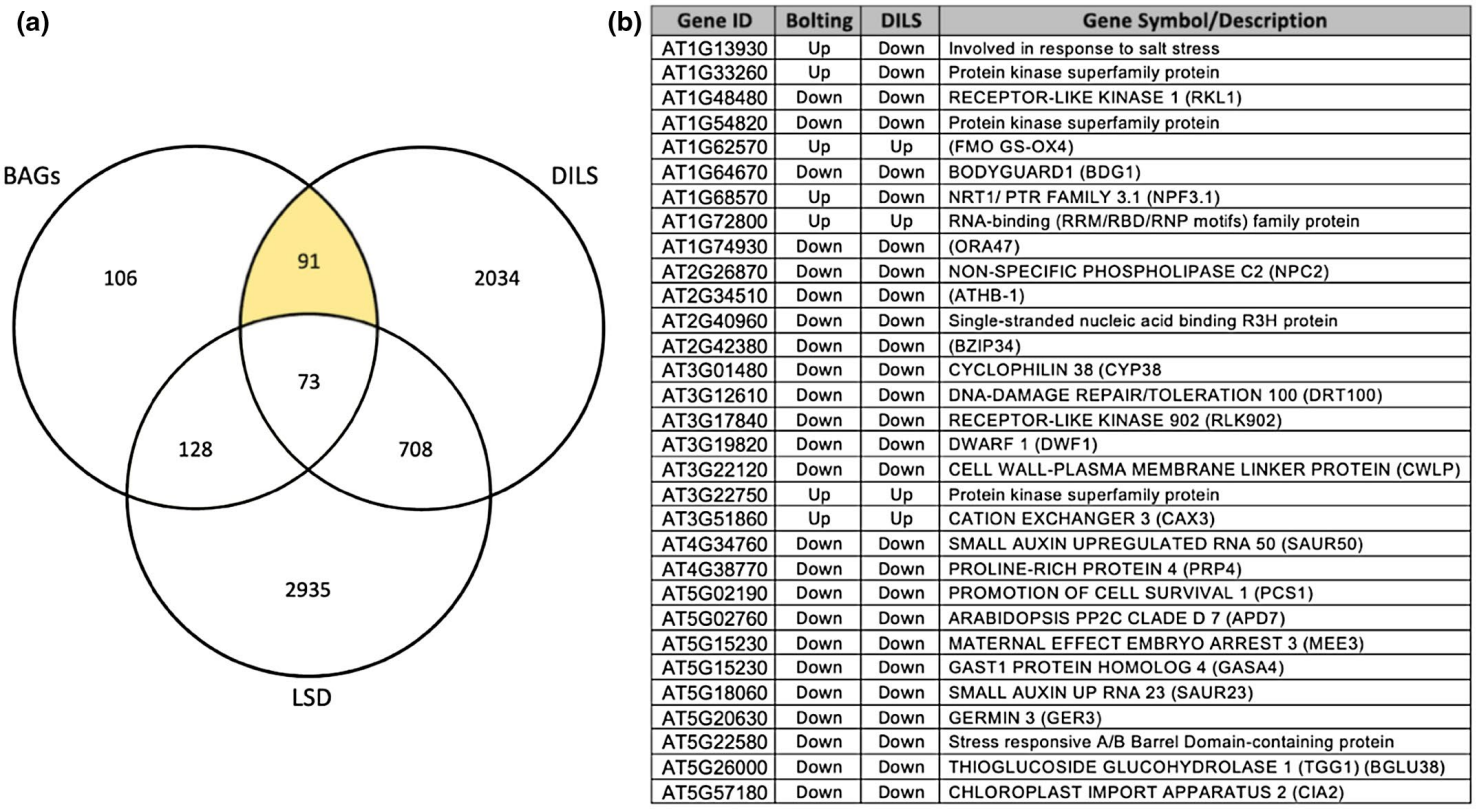
Potential novel LS regulators. The Venn Diagram shows overlap between the 398 BAGs, the 2906 DEGs from the DILS dataset, and the 3844 SAGs in the Leaf Senescence Database (LSD 3.0). The 91 BAGs that were differentially expressed in the DILS dataset but not in the LSD 3.0 are highlighted. Select genes from this list of 91 genes are shown in the table along with their direction of gene expression in bolting *atx1,3,4* TMs and during DILS. Gene Symbols/Descriptions were obtained using the bulk data retrieval tool in TAIR

We intersected the DILS day 3 DEGs with the BAG and LSD 3.0 gene lists, and a significant overlap was found (*p* < .001) (Figure 7). A total of 91 BAGs were shared in the DILS experiment, but were not in the LSD 3.0 (Figure 7a, Data File S4, Sheet 8). Sixty‐eight (74.7%) of these 91 genes were downregulated during *atx1,3,4* TM bolting and DILS, while 15 (16.5%) were upregulated during *atx1,3,4* TM bolting and DILS. Seven of these 91 genes were upregulated in bolting *atx1,3,4* TMs, but downregulated during DILS; and one gene was downregulated during *atx1,3,4* TM bolting, but upregulated during DILS. The 91 BAGs that change expression during DILS, but are not present in LSD 3.0, are genes that may represent novel early regulators of LS (Figure 7b).

### 3.6 Bolting‐associated gene regulatory network (GRN)

**FIGURE 8:**
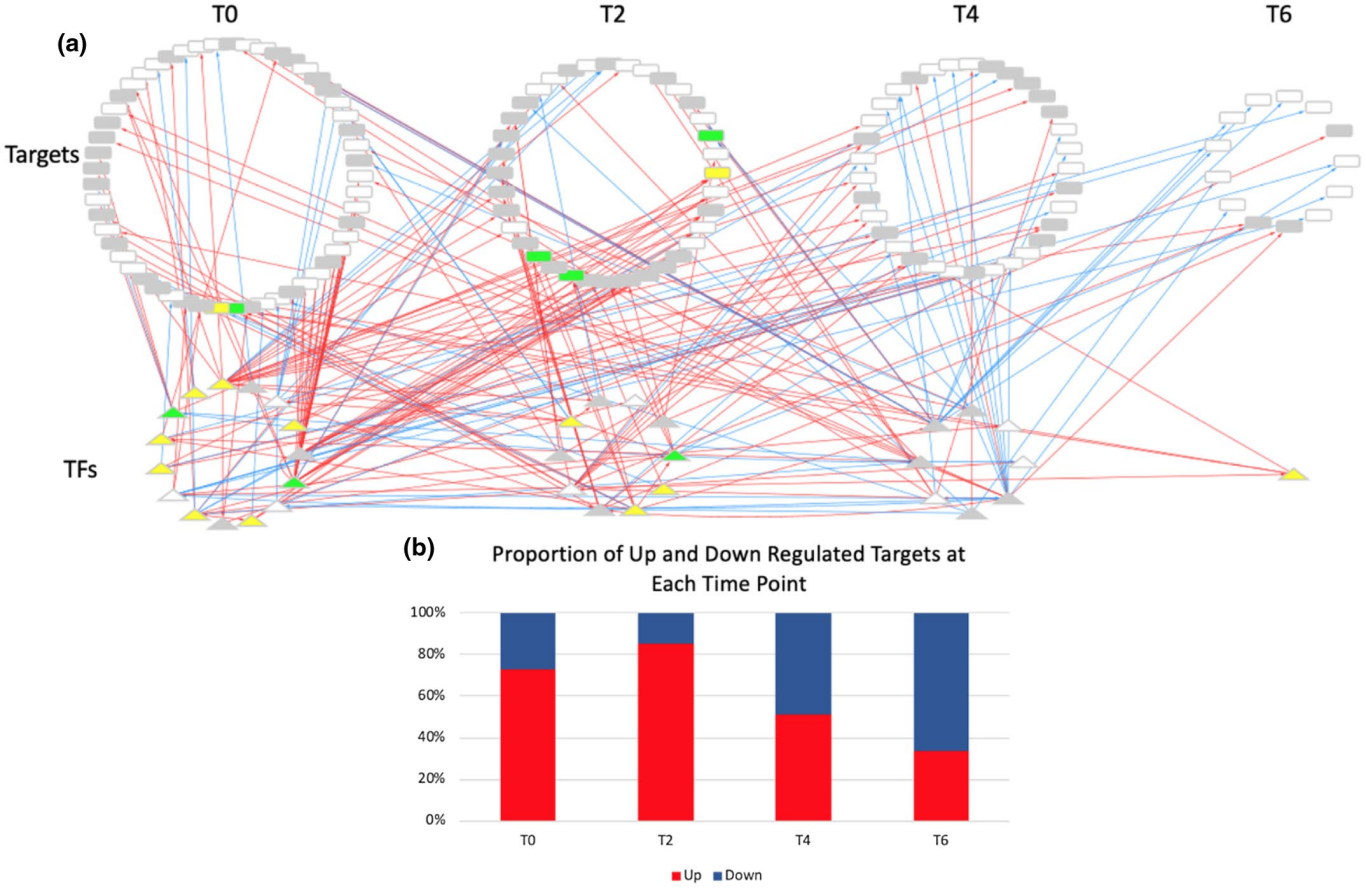
Bolting time‐associated gene regulatory network. (a) The network was constructed as described in the methods and uploaded to Cytoscape for visualization. White nodes were not present in the LSD 3.0. Grey nodes were present, but had an unclear function in LS. Yellow nodes promote LS while green nodes prevent LS. Upregulation is shown by red edges and downregulation is shown with blue edges. Node shape refers to the type node, where triangles are TFs, and rectangles are not TFs. (b) The proportion of upregulated versus downregulated target genes at each time point is shown.

We sought to identify genetic interactions between BAGs, of which many are transcription factors (TFs). We employed a machine learning approach (GENIE3) to build a network and then trimmed it with a precision‐recall analysis using DAP‐seq binding data in ConnecTF.org (Huynh‐Thu et al., 2010; Juang et al., 2020) (Figure S2a). We then annotated the network by time, node type, direction of expression at bolting and during DILS, and annotation in the LSD 3.0 (Data File S7 contains the fully annotated network). These data were uploaded to Cytoscape for visualization (Shannon et al., 2003). To reduce network density, nodes are only shown at the first time they were differentially expressed. A side effect of this display is that some interactions appear reversed in time. An example of this can be seen with the T2 TFs regulating targets at T0 (Figure 8).

**FIGURE 9:**
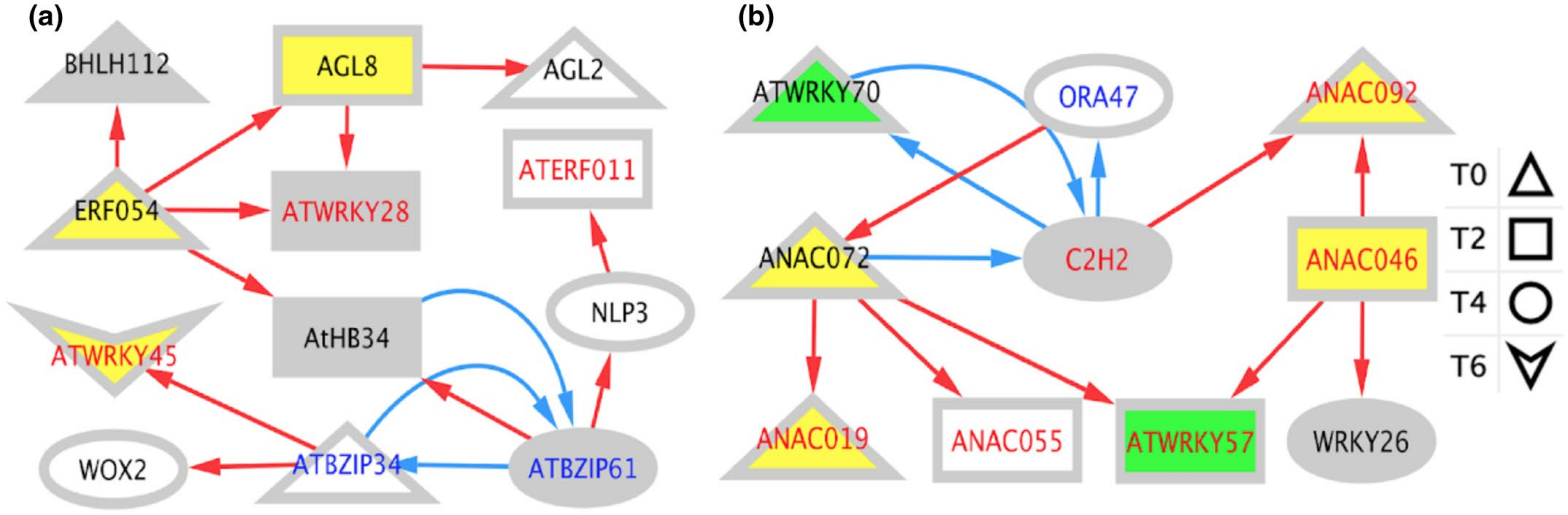
Time‐resolved TF interaction networks. (a) A transcriptional cascade downstream of ERF054 is shown. (b) A NAC TF Family centric network is shown. All nodes in these networks are TFs. Node shape refers to the first time of differential expression for that particular TF. Node color refers to annotation in LSD 3.0. White nodes were not present in the LSD. Grey nodes were present, but had an unclear function in LS. Yellow nodes promote LS while green nodes prevent LS. Edge color refers to the direction of gene expression in flowering *atx1,3,4* TMs relative to vegetative WT Col‐0 plants. Upregulation is shown by red edges and downregulation is shown with blue edges. Label color corresponds to direction of TF gene expression during DILS, with red indicating upregulation and blue indicating downregulation. For example, nodes with red edges and red labels represent TFs that are upregulated at bolting and during DILS. Black labels mark nodes that were not differentially expressed during DILS

**FIGURE 10:**
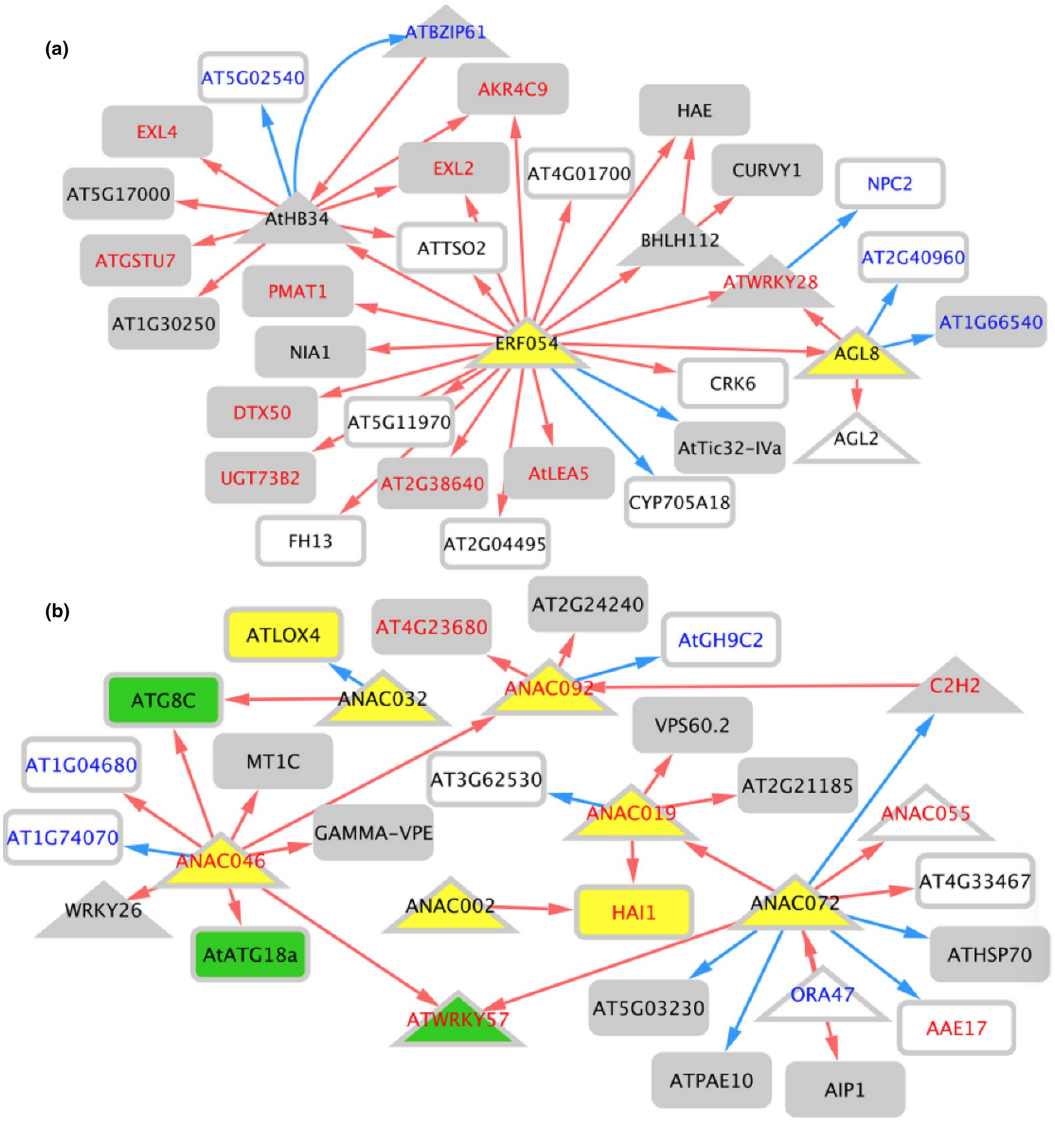
Select steady‐state TF‐target subnetworks. (a) Predicted targets of ERF054 are shown. (b) Predicted targets downstream of NAC TFs are shown. These networks are not time resolved. Node shape refers to the node type: Triangles are TFs while rectangles are not TFs. Node color refers to annotation in the LSD 3.0. White nodes were not present in the LSD. Grey nodes were present, but had an unclear function in LS. Yellow nodes promote LS while green nodes prevent LS. Edge color refers to the direction of gene expression in flowering *atx1,3,4* TMs relative to vegetative WT Col‐0 plants. Upregulation is shown by red edges and downregulation is shown with blue edges. Label color corresponds to direction of TF gene expression during DILS, with red indicating upregulation and blue indicating downregulation. For example, nodes with red edges and red labels represent TFs that are upregulated at bolting and during DILS. Potential Novel LS regulators are shown as genes not included in the LSD 3.0 (white nodes) that are DILS DEGS (red or blue labels). Black labels mark nodes that were not differentially expressed during DILS

All nodes (TFs and Targets) in the network are BAGs (Figures 8a, 9, and 10). The network shows that there are bolting time‐associated TFs that can bind to and may cause the differential expression of target BAGs. There is a shift from upregulation (red) to downregulation (blue) from T0 to T6 (Figure 8b). While many BAG TFs act independently, some interactions between TFs were identified (Figure 9). Select genes were included in the subnetworks highlighting targets genes downstream of *ERF054* and the NAC TFs (Figure 10). All TFs in the subnetworks confer differential expression at multiple time points and have both shared and independent targets.

The time‐resolved TF networks show integration of LS‐related signaling (*ERF054, WRKY28, WRKY45*) with flowering signaling (*AGL8, AGL2/SEP1*) (Figure 9a). Most TFs that change expression during DILS change expression in the same direction at bolting time, for example, red edges leading to nodes with red labels (Figure 9a,b). This is also true for most potential novel early regulators of LS, which are the genes that are differentially expressed during DILS but are not in LSD 3.0 (nodes with white backgrounds with red or blue labels).

## 4 DISCUSSION

The goal of our study was to better understand the molecular connection between the vegetative‐reproductive transition and LS in Arabidopsis. We hypothesized that there are LS‐related gene expression changes associated with the bolting event. Using RNA‐seq, we generated a list of 398 bolting‐associated genes (BAGs). A total of 202 of these BAGs were present in the Leaf Senescence Database 3.0 (LSD 3.0), some of which may be responsible for temporally connecting LS to bolting time. We also identified 91 BAGs that are differentially expressed during dark‐induced leaf senescence (DILS) but are not present in the LSD 3.0. Further study of these 91 genes may reveal some novel early regulators of LS.

### 4.1 *atx1,3,4* Triple‐mutant phenotypes

By mutating *ATX* genes, we engendered early flowering by altering the expression of known flowering time regulators *FLC, SOC1*, and *FT*. The small change in flowering time (5–7 days) was advantageous compared to studying an extreme flowering time phenotype that could have added a prolonged age bias to the experiment. Furthermore, we could not use stress‐induced early flowering of WT because stress and LS signaling overlap. *atx1,3,4* TM mutants show no visible signs of stress or other developmental defects prior to or after bolting.

By overlapping the results between two separate *atx1,3,4* isolates, TM1 and TM2, we reduced the probability of false discoveries. Early flowering has been reported in other *atx* mutants (Berr et al., 2015; Yun et al., 2012), which is consistent with our findings. Chen et al isolated an (*atx3, atx4, atx5)* triple mutant but did not report a change in flowering time or LS (Chen, Luo, et al., 2017). The five *ATX* enzymes are classified into two clades (*ATX1* and *ATX2*) and (*ATX3, ATX4*, and *ATX5)*, thus, their genetic divergence may explain the difference in phenotypes between the (*atx1 atx3 atx4*) and the (*axt3 atx4 atx5*) triple mutants.

### 4.2 RNA‐seq time‐series experiment

Synchronizing tissue harvesting to bolting differentiated bolting‐associated and age‐associated changes in gene expression. Multiple statistical approaches were used for the identification of 750 initial DEGs since there is not one perfect statistical approach for our time‐series analysis. Treating time as a continuous variable as we did in DESeq2 helps to identify time‐resolved gene expression changes. However, our edgeR‐based method treated time as a factor, which may have allowed greater detection of transient changes in gene expression. It is common to see high overlap between edgeR and DESeq2, however, that typically occurs when the same underlying statistical design is used. Here, we did not expect strong overlap as the programs were run with different designs. Even with the varying designs, Genesect found significant overlap between these gene lists (*p* < .001).

Lack of reproducibility for large dataset analysis is a chronic issue (Łabaj & Kreil, 2016; Simoneau et al., 2019). While some programs are commonly used for differential expression analysis (edgeR, DESeq2, limma), we felt justified to include a *T* test method, as it increased transparency and showed that a bulk of the DEGs identified by the two commonly used programs were supported by simple *T* tests. Our stringent overlapping method should reduce false positives.

It was also important to validate our results in WT plants because the *atx1,3,4* TMs may have unknown epigenetic effects. Ideally, the RNA‐seq time‐course experiment would be repeated centering on bolting time in WT plants with a late‐flowering vegetative control. As a substitute, public data were more practical and cost‐effective. While this is a limitation to our study, the two published experimental designs were similar to ours. These two WT datasets used similarly aged leaves, similar time resolution, and were centered around the vegetative‐reproductive transition, which was made apparent by the identification of *FT* and *SOC1* as DEGs in both. As expected, our dataset had stronger overlap with Breeze *et al*. than Del Prete *et al*., as plants in the Breeze *et al*. dataset were bolting. Del Prete *et al*. was specifically looking at the genetic changes associated with *FT* and *SOC1* expression, and their plants were not yet bolting. Both datasets had multiple replicates for high confidence output. Ultimately, changes in gene expression need to be validated with real‐time qPCR of WT biological replicates.

We are also confident that our time resolution was adequate to detect bolting‐associated gene expression changes, although we cannot rule out an age‐dependent contribution, as shown by the transcriptome PCA (Figure 3b). T6 WT plants were nearing the vegetative transition, and one WT plant had bolted in our RNA‐seq experiment. We argue that this is a strength of our design, as it allows us to further show that our DEGs are likely associated with bolting. For example, *WRKY45* is induced at T0 in *atx1,3,4* TMs while it is maintained at very low levels in WT. At T6, however, there is a slight increase in *WRKY45* expression in WT as plants near the vegetative transition, which further supports our claim that its change in expression is likely associated with the bolting event. In both RNA‐seq experiments, *WRKY45* is not expressed until after the induction of flowering, meaning the induction of *WRKY45* gene expression may specifically be associated with phase transition signaling (Figure 5). *DTX50* is specifically induced 4 days after bolting in both our experiment and in the Breeze et al experiment, showing how using datasets with similar time resolution helped confirm gene expression changes (Figure 5).

### 4.3 Bolting‐associated genes (BAGs)

The 398 BAGs were enriched for LS and LS‐related biological process GO terms. This supports the hypothesis that bolting stimulates LS‐related signaling in mature leaves. The following known LS‐associated genes were identified in our *atx1,3,4* TM analysis, and then further validated in both WT time‐series experiments. *SOC1* is a classic example of a gene that positively regulates both flowering time and LS. *ANAC032* positively regulates flowering time and LS and is responsive to oxidative stress (Mahmood et al., 2016). *SAG20* and *SEN1* are both LS markers (Fernández‐Calvino et al., 2016; Schenk et al., 2005; Weaver et al., 1998). *DEAR1* promotes cell death and SA synthesis (Tsutsui et al., 2009), while *DTX50* is a transiently expressed ABA transporter (Zhang et al., 2014). The induction of *DTX50* soon after bolting time in all experiments indicates ABA flux could be associated with bolting. *ADP7 (SSPP)* is a senescence suppressed protein phosphatase (Xiao et al., 2015).

Other LS‐related BAGs were shared between the *atx1,3,4* TMs and Breeze *et al*. dataset. *ORE1* (*ANAC092*) is a well‐studied promoter of LS (Kim, Kim, et al., 2018; Qiu et al., 2015). *WRKY28* is responsible for activating SA biosynthesis genes and promotes LS (Tian et al., 2020; van Verk et al., 2011). *SAG13* is a ROS‐responsive BAG that regulates DILS (Dhar et al., 2020). When knocked out in higher‐order mutants to reduce redundancy with other CRF TFs, *crf2* mutants showed delayed LS, suggesting the BAG *CRF2* works with other CRF TFs to promote LS (Raines et al., 2016). *BOI* attenuates cell death and regulates flowering time (Nguyen et al., 2015), while BHLH112 plays roles in both flowering time and stress response (Chen, Hsieh‐Feng, et al., 2017; Liu et al., 2015).

The following genes were shared between the *atx1,3,4* TMs and the Del Prete et al. dataset. *WRKY46* is responsible for activating SA biosynthesis genes and promoting LS (van Verk et al., 2011). *WRKY48* is a stress and pathogen‐induced regulator of plant defense (Xing et al., 2008). The BAG *SAUR41* acts redundantly with *SAUR49* to promote LS by regulating *SSPP*, another BAG with a known role in LS (Wen et al., 2020).

Other BAGs associated with flowering time, seed development, and seed nutrient content were also identified. *UMAMI28* is responsible for transporting amino acids to the developing seeds (Müller et al., 2015), and *SUS3* regulates sugar metabolism in developing seeds (Angeles‐Núñez & Tiessen, 2010). The BAG *SEP1* works redundantly with *SEP2* and *SEP3* to regulate flower and ovule development (Pelaz et al., 2000). *CURVY1* regulates both flowering time and seed development (Gachomo et al., 2014). *FUL (AGL8)* and *BFT* regulate flowering time (Balanzà et al., 2014; Bemer et al., 2017; Ryu et al., 2011, 2014). These genes change expression in the 6th rosette leaf, suggesting they may contribute to processes other than flower and seed development and seed nutrition.

### 4.4 Potential novel LS regulators

Ninety‐one BAGs were not present in LSD 3.0, but were differentially regulated during DILS (Figure 7b). DILS and age‐dependent LS have different signaling stimuli, but over time, the pathways become shared (Guo & Gan, 2012). We chose the last available time point in the time series (3 days) as we felt it would be most similar to the LS observed in our RNA‐seq. Many well‐characterized age‐dependent LS regulators are in the DILS DEG list, supporting it as an appropriate estimation of LS signaling. A majority of the potential novel LS regulators (91%) change expression in the same direction during both bolting and DILS, but seven (7.7%) are upregulated at bolting and downregulated during DILS. Further study may show that some of these seven genes might prevent LS prior to and during bolting, and then must be downregulated for normal or dark‐induced LS to proceed. Some of these 91 genes regulate processes that are known to be related to LS. For example, *ORA47* regulates both ABA and JA synthesis (Chen, Hsieh, et al., 2016). While some of these 91 genes have known functions related to LS, a phenotype screen of verified mutants is needed to support their function.

### 4.5 GRNs

There is a shift from upregulation to downregulation in the BAG network (Figure 8). This is consistent with the findings of the NAC troika, the LS regulatory hub that prevents precocious LS early in plant development. The time‐resolved network downstream of ERF054 shows a net positive regulation of LS (Figure 9a). Overexpression of *ERF054* was found to promote LS, which is consistent with the structure of our network (Xu et al., 2010). *WRKY45* is a TF downstream of ERF054 that has been shown to promote both flowering time and LS (Chen, Xiang, et al., 2017). ERF054 is also predicted to be upstream of genes related to flowering time (*AGL2/SEP1*, *AGL8*), nitrogen signaling and metabolism (*NLP3, NIA1*), and stress and LS signaling (*LEA5/SAG21, DTX50, WRKY28*) (Figures 9 and 10) (Gu et al., 1998; Mohn et al., 2019; Olas & Wahl, 2019; Pan et al., 2019; Pelaz et al., 2000; Salleh et al., 2012; van Verk et al., 2011; Zhang et al., 2014). *ERF054* may be a candidate regulatory gene that links stress, LS, nitrogen metabolism, and flowering time signaling. Further genetic analysis has been hampered by the inability to isolate an *erf054* T‐DNA insertion knockout mutant.

Furthermore, BZIP61 and BZIP34 (Figure 9a) share 71% amino acid identity and are known to form heterodimers to regulate pollen development (Gibalová et al., 2009). Their differential expression downstream of ERF054 in leaf tissue may indicate a novel regulatory role unrelated to pollen development. We intend to generate a double knockout mutant to test this hypothesis. The NAC network contains many well‐characterized regulators of LS, including a regulatory hub of three NAC TFs that control LS (*ANAC055, ANAC019, ANAC072*) (Hickman et al., 2013). Other TFs in the network also have known roles in LS (*WRKY57, ANAC046, WRKY70, ANAC092/ORE1*) (Jiang et al., 2014; Kim, Park, et al., 2018; Oda‐Yamamizo et al., 2016; Ülker et al., 2007).

## 5 CONCLUSION

We have identified 398 BAGs expressed in mature leaf 6 that change expression at the time of bolting as the plant ages. A total of 202 of these BAGs are known to be associated with LS as demonstrated by their presence in LSD 3.0 (Li et al., 2020). Together, this study identifies LS‐related gene expression changes that occur in a specific mature rosette leaf after the vegetative to reproductive transition at the shoot apical meristem. Further study may reveal that some of these LS‐related BAGs contribute to the temporal relationship between flowering time and LS.

## CONFLICT OF INTEREST

The authors declare no conflict of interest associated with the work described in this manuscript.

## AUTHOR CONTRIBUTIONS

JAB and WEH designed the experiments. WEH completed the experiments and data analyses. WEH and JAB wrote and edited the manuscript.

## ACCESSION NUMBERS

RNA‐seq data from this study are publicly available on GEO as GSE134177.

## STATEMENT OF CONTRIBUTIONS TO FIELD

While it is widely accepted that early bolting generally confers early leaf senescence in Arabidopsis, the molecular basis of this temporal relationship has not been explored. Toward a molecular definition of this relationship, leaf senescence‐related gene expression changes were identified in mature rosette leaves at the time of bolting. Further study may show that some of these contribute to the temporal coupling of LS and bolting. This information could help inform the production of crops that could overcome early leaf senescence during stress‐induced early bolting.

## Supporting information

Fig S1

Fig S2

DataFIile S1

DataFIile S2

DataFIile S3

DataFIile S4

DataFIile S5

DataFIile S6

DataFIile S7
