# Supplementary material for: Gene expression changes occurring at bolting time are associated with leaf senescence in Arabidopsis
Source: Plant Direct. 2020 Nov 8;4(11):e00279. doi: 10.1002/pld3.279 (PMC7649007; doi:10.1002/pld3.279)
Supplement: Supplementary file 2 — Fig S2 [file PLD3-4-e00279-s002.docx]

**
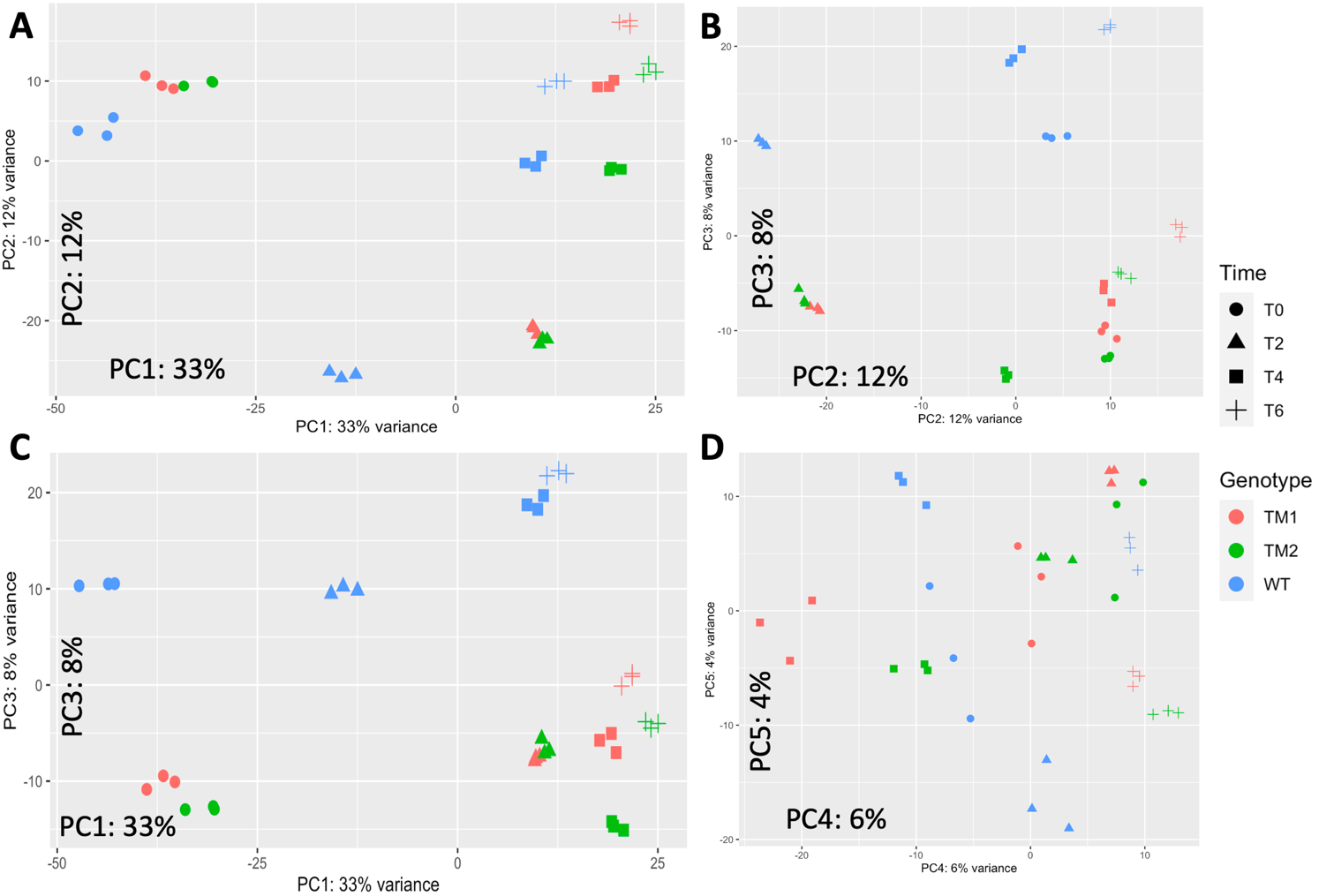
**

**Supplemental Figure 2A: Extended PCA analysis**

PCA run on entire transcriptomes was completed using an amended plotPCA function in DESeq2. All replicates cluster close together. PC1 separates samples by time, (see panel 2A-C). PC2 separates T2 from other time points. PC3 clearly separates samples by genotype/bolting phenotype (see panel 2A-B and 2A-C). Time, Genotype, and Phenotype, were captured by PC1, PC2, and PC3. PC4 and PC5 do not separate data by any meaningful patterns, suggesting PC1 through PC3 sufficiently describe the data. (panel 2A-D).


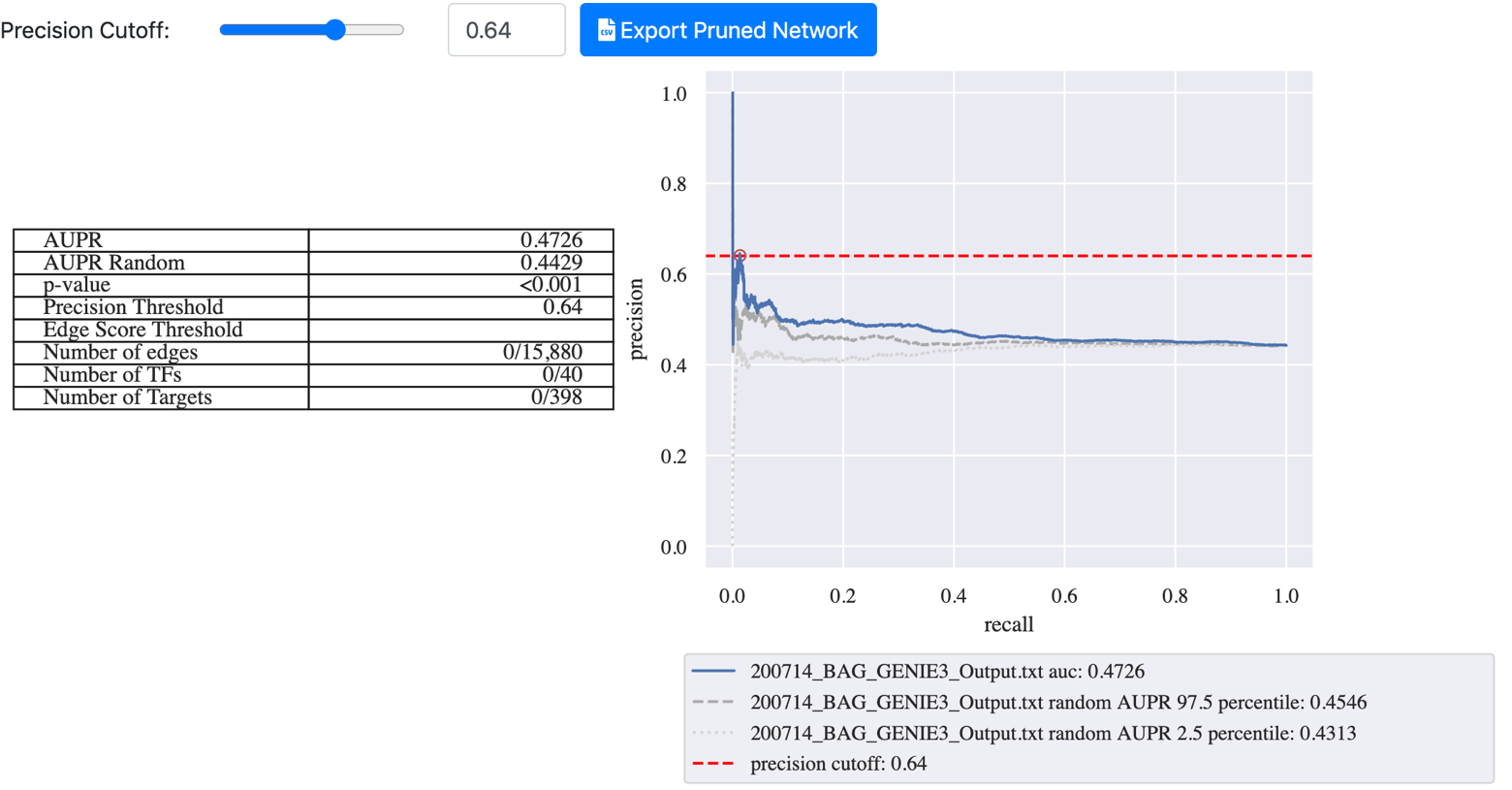
 **Supplemental Figure 2B: AUPR Analysis from ConnecTF.org**

Using DAP-seq binding data in ConnecTF.org, we found that our GENIE3-predicted network performed better than random (shown by grey lines). There was a high peak of increased precision, marked by the red cutoff line. There was also a steady improvement in recall. Together these show our machine learning performed well and our interactions were generally well supported by DAP-seq TF binding data. We selected the highest precision cutoff (in relation to random) as a network pruning cutoff to increase the stringency of the interactions shown in Figures 8-10.
