# Supplementary material for: Gene expression changes occurring at bolting time are associated with leaf senescence in Arabidopsis
Source: Plant Direct. 2020 Nov 8;4(11):e00279. doi: 10.1002/pld3.279 (PMC7649007; doi:10.1002/pld3.279)
Supplement: Supplementary file 5 — DataFIile S3 [file PLD3-4-e00279-s005.docx]

***DESeq2: LRT,***

if (!requireNamespace("BiocManager", quietly = TRUE))

install.packages("BiocManager")

##Just TM1, repeat with Tm2,then overlap the DEG lists

library(DESeq2)

#subset just the numerical data (remove the AT numbers)

mydata <- as.data.frame(X200322_T1Counts[c(2:25)])

#add the AT numbers back in as row names

row.names(mydata) <- X200322_T1Counts$target_id

#Round the data

mydata<-round(mydata)

#Define experimental design

T1dds <- DESeqDataSetFromMatrix(countData = mydata,

colData = X200322_T1ES,

design= ~ ~ Genotype + Time+Genotype:Time)

#remove genes with consistently low expression

nrow(T1dds)

keep <- rowSums(counts(T1dds)) > 1

T1dds <- T1dds[keep,]

nrow(T1dds)

#Run the DE analysis

T1dds <- DESeq(T1dds, test="LRT", reduced = ~ Time)

#Return Results, degs different between genotypes

resT1 <- results(T1dds, contrast=c("Genotype","WT","Mutant"))

mcols(resT1, use.names = TRUE)

summary(resT1)

#Subset genes with p<.05 and FDR<.05

T1Sig <- subset(resT1,abs(padj)< 0.05)

write.csv(T1Sig, file = "200325T1DeSEQ2.csv")

##This is identical to the previous chunk of code, just repeated for the second mutant

library(DESeq2)

mydata1 <- as.data.frame(X200322_T2Counts[c(2:25)]) #subset just the numerical data (remove the AT numbers)

row.names(mydata1) <- X200322_T2Counts$target_id #add the AT numbers back in as row names

mydata1<-round(mydata1)

T2dds <- DESeqDataSetFromMatrix(countData = mydata1,

colData = X200322_T2ES,

design= ~ ~ Genotype + Time+Genotype:Time)

nrow(T2dds)

keep <- rowSums(counts(T2dds)) > 1

T2dds <- T2dds[keep,]

nrow(T2dds)

T2dds <- DESeq(T2dds, test="LRT", reduced = ~ Time)

resT2 <- results(T2dds, contrast=c("Genotype","WT","Mutant"))

mcols(resT2, use.names = TRUE)

summary(resT2)

T2Sig <- subset(resT2,abs(padj)< 0.05)

write.csv(T2Sig, file = "200325T1DeSEQ2.csv")

T1AT<-as.data.frame(rownames(T1Sig))

T2AT<-as.data.frame(rownames(T2Sig))

#overlap the DEG lists from T1 and T2

DESeq2DEGs<- as.data.frame(intersect(T1AT$`rownames(T1Sig)`,T2AT$`rownames(T2Sig)`))

write.csv(DESeq2DEGs, file = "200325DESeq2DEGList.csv")

***#edgeR model:***

library(edgeR)

library(limma)

library(RColorBrewer)

library(HTSFilter)

#TM1

#subset just the numerical data (remove the AT numbers)

mydata <- as.data.frame(X200322_T2Counts[c(2:25)])

#add the AT numbers back in as row

row.names(mydata) <- X200322_T2Counts$target_id

#define the groups for design (combo contains genotype and time in one string (WT0,WT2, etc)

group<-X200322_T2ES$Combo

#make matrix

y <- DGEList(counts=(mydata), group=group)

#remove genes with low expression

keep <- filterByExpr(y)

y <- y[keep, , keep.lib.sizes=FALSE]

#calculate the library sizes for normalization

y <- calcNormFactors(y)

#Read to make sure it worked and check the groups look right

y$samples

#Assign the groups to the design matrix, to group which samples will be tested together

design <- model.matrix(~0+group)

#Define what comparisons to make, here I assign WT at time point 0 to mutant at time point 0

my.contrasts <- makeContrasts(

T0 = groupWT0-groupMut0,

T2 = groupWT2-groupMut2,

T4 = groupWT4-groupMut4,

T6 = groupWT6-groupMut6,

levels=design)

#estimate dispersion, I think this finds variance of mean relative to library size per gene?

y <- estimateDisp(y, design)

#combine the design and count matrix data.

fit <- glmQLFit(y, design)

#Run the tests

T1T0 <- glmQLFTest(fit, contrast=my.contrasts[,"T0"])

T1T2 <- glmQLFTest(fit, contrast=my.contrasts[,"T2"])

T1T4 <- glmQLFTest(fit, contrast=my.contrasts[,"T4"])

T1T6 <- glmQLFTest(fit, contrast=my.contrasts[,"T6"])

#isolate transcripts with p<.05 and a logFC greater than 1.5

T1T0Sig<-subset(T1T0$table, PValue<.05)

T1T2Sig<-subset(T1T2$table, PValue<.05)

T1T4Sig<-subset(T1T4$table, PValue<.05)

T1T6Sig<-subset(T1T6$table, PValue<.05)

T1T0SigLog<-subset(T1T0Sig, abs(logFC)>1.5)

T1T2SigLog<-subset(T1T2Sig, abs(logFC)>1.5)

T1T4SigLog<-subset(T1T4Sig, abs(logFC)>1.5)

T1T6SigLog<-subset(T1T6Sig, abs(logFC)>1.5)

#get transcript names

T1T0SigAT<-as.data.frame(rownames(T1T0SigLog))

T1T2SigAT<-as.data.frame(rownames(T1T2SigLog))

T1T4SigAT<-as.data.frame(rownames(T1T4SigLog))

T1T6SigAT<-as.data.frame(rownames(T1T6SigLog))

write.csv(T1T0SigAT, file = "edgeRT2-0Sig.csv")

write.csv(T1T2SigAT, file = "edgeRT2-2Sig.csv")

write.csv(T1T4SigAT, file = "edgeRT2-4Sig.csv")

write.csv(T1T6SigAT, file = "edgeRT2-6Sig.csv")

I repeated this identical code with TM2, then reported the intersection between TM1 vs. WT and TM2 vs. WT.

T-Test based approach:

library (vegan)

library (Hmisc)

library (ggplot2)

library(reshape)

library(reshape2)

library(splitstackshape)

library(plyr)

library(gdata)

ncol(FPKMafterfilter1) #how many columns (should be 36, but 37 with labels)

mydata <- as.data.frame(FPKMafterfilter1[c(2:37)]) #subset just the numerical data (remove the AT numbers)

row.names(mydata) <- FPKMafterfilter1$X1 #add the AT numbers back in as row names

#everything below separates out samples by replicates

W0 <- as.data.frame(mydata[c(1:3)])

W2 <- as.data.frame(mydata[c(4:6)])

W4 <- as.data.frame(mydata[c(7:9)])

W6 <- as.data.frame(mydata[c(10:12)])

T10 <- as.data.frame(mydata[c(13:15)])

T12 <- as.data.frame(mydata[c(16:18)])

T14 <- as.data.frame(mydata[c(19:21)])

T16 <- as.data.frame(mydata[c(22:24)])

T20 <- as.data.frame(mydata[c(25:27)])

T22 <- as.data.frame(mydata[c(28:30)])

T24 <- as.data.frame(mydata[c(31:33)])

T26 <- as.data.frame(mydata[c(34:36)])

#get the average of three replicates in each file

W0mean <- as.data.frame(rowMeans(W0[,1:3]))

W2mean <- as.data.frame(rowMeans(W2[,1:3]))

W4mean <- as.data.frame(rowMeans(W4[,1:3]))

W6mean <- as.data.frame(rowMeans(W6[,1:3]))

T10mean <- as.data.frame(rowMeans(T10[,1:3]))

T12mean <- as.data.frame(rowMeans(T12[,1:3]))

T14mean <- as.data.frame(rowMeans(T14[,1:3]))

T16mean <- as.data.frame(rowMeans(T16[,1:3]))

T20mean <- as.data.frame(rowMeans(T20[,1:3]))

T22mean <- as.data.frame(rowMeans(T22[,1:3]))

T24mean <- as.data.frame(rowMeans(T24[,1:3]))

T26mean <- as.data.frame(rowMeans(T26[,1:3]))

#calculate fold change for TM over WT.

T10Fold1<- as.data.frame((T10mean/W0mean))

T20Fold1<- as.data.frame((T20mean/W0mean))

T12Fold1<- as.data.frame((T12mean/W2mean))

T22Fold1<- as.data.frame((T22mean/W2mean))

T14Fold1<- as.data.frame((T14mean/W4mean))

T24Fold1<- as.data.frame((T24mean/W4mean))

T16Fold1<- as.data.frame((T16mean/W6mean))

T26Fold1<- as.data.frame((T26mean/W6mean))

#rename the columns to say "Fold"

names(T10Fold1)[1] <- "Fold"

names(T20Fold1)[1] <- "Fold"

names(T12Fold1)[1] <- "Fold"

names(T22Fold1)[1] <- "Fold"

names(T14Fold1)[1] <- "Fold"

names(T24Fold1)[1] <- "Fold"

names(T16Fold1)[1] <- "Fold"

names(T26Fold1)[1] <- "Fold"

#Creating files for sig data to be put into

test2 <- NULL

test4<- NULL

#Overall ttest: sta is needed for directionality, so the data can be subset to up or downregulated for fold change calcs

#T0, WT vs TM1 and TM2

for (j in seq(nrow(W0)))

{

ID_REF<-rownames(W0[j,]) #to extract the probe id

sta1<-t.test(W0[j,], T10[j,])$statistic #to run the t.test and extract the $statistic (i.e., the difference in the mean vale)

sig1<-t.test(W0[j,], T10[j,])$p.value #to (re)run the t.test and extract the significance value (i.e., p-value)

test1<-cbind(ID_REF,sta1,sig1) # this juxtaposing sta and sig

test2<-rbind(test2,test1) # to same sta and sig

ID_REF<-rownames(W0[j,]) #to extract the probe id

sta2<-t.test(W0[j,], T20[j,])$statistic #to run the t.test and extract the $statistic (i.e., the difference in the mean vale)

sig2<-t.test(W0[j,], T20[j,])$p.value #to (re)run the t.test and extract the significance value (i.e., p-value)

test3<-cbind(ID_REF,sta2,sig2) # this juxtaposing sta and sig

test4<-rbind(test4,test3) # to same sta and sig

}

#Add Fold Change Data into it

head(test2)

test2<- as.data.frame(test2)

test2$Fold<- T10Fold1

#make a numeric dataset

test2$sta1<-as.numeric(as.character(test2$sta1))

test2$sig1<-as.numeric(as.character(test2$sig1))

#isoalte the significant results with pvalue of .05 and a 2 fold change

sig_wt_T1_0 <- subset(test2,abs(sig1)<.05)

sig2foldT101 <- subset(sig_wt_T1_0,abs(Fold)>2)

sig2foldT102 <- subset(sig_wt_T1_0,abs(Fold)<0.5)

T1_0_Sig <- combine(sig2foldT101,sig2foldT102)

#remove excess files so they dont stack up

rm(sig_wt_T1_0)

rm(sig2foldT101)

rm(sig2foldT102)

rm(sta1)

rm(sig1)

rm(test1)

rm(test2)

#repeat for TM2

test4<- as.data.frame(test4)

test4$Fold<- T20Fold1

#make a numeric dataset

test4$sta2<-as.numeric(as.character(test4$sta2))

test4$sig2<-as.numeric(as.character(test4$sig2))

#isoalte the significant results with a bonferonni correction., .05/19437=.00000257

sig_wt_T2_0 <-subset(test4,abs(sig2)<.05)

sig2foldT201 <-subset(sig_wt_T2_0,abs(Fold)>2)

sig2foldT202 <- subset(sig_wt_T2_0,abs(Fold)<0.5)

T2_0_Sig <- combine(sig2foldT201,sig2foldT202)

rm(sig_wt_T2_0)

rm(sig2foldT201)

rm(sig2foldT202)

rm(sta2)

rm(sig2)

rm(test3)

rm(test4)

#start for the next time point

test6 <- NULL

test8<- NULL

#T2, WT vs TM1 and TM2

for (j in seq(nrow(W2)))

{

ID_REF<-rownames(W2[j,]) #to extract the probe id

sta3<-t.test(W2[j,], T12[j,])$statistic #to run the t.test and extract the $statistic (i.e., the difference in the mean vale)

sig3<-t.test(W2[j,], T12[j,])$p.value #to (re)run the t.test and extract the significance value (i.e., p-value)

test5<-cbind(ID_REF,sta3,sig3) # this juxtaposing sta and sig

test6<-rbind(test6,test5) # to same sta and sig

ID_REF<-rownames(W2[j,]) #to extract the probe id

sta4<-t.test(W2[j,], T22[j,])$statistic #to run the t.test and extract the $statistic (i.e., the difference in the mean vale)

sig4<-t.test(W2[j,], T22[j,])$p.value #to (re)run the t.test and extract the significance value (i.e., p-value)

test7<-cbind(ID_REF,sta4,sig4) # this juxtaposing sta and sig

test8<-rbind(test8,test7) # to same sta and sig

}

test6<- as.data.frame(test6)

test6$Fold<- T12Fold1

#make a numeric dataset

test6$sta3<-as.numeric(as.character(test6$sta3))

test6$sig3<-as.numeric(as.character(test6$sig3))

sig_wt_T1_2 <-subset(test6,abs(sig3)<.05)

sig2foldT121 <-subset(sig_wt_T1_2,abs(Fold)>2)

sig2foldT122 <- subset(sig_wt_T1_2,abs(Fold)<0.5)

T1_2_Sig <- combine(sig2foldT121,sig2foldT122)

test8<- as.data.frame(test8)

test8$Fold<- T22Fold1

#make a numeric dataset

test8$sta4<-as.numeric(as.character(test8$sta4))

test8$sig4<-as.numeric(as.character(test8$sig4))

sig_wt_T2_2 <-subset(test8,abs(sig4)<.05)

sig2foldT221 <-subset(sig_wt_T2_2,abs(Fold)>2)

sig2foldT222 <- subset(sig_wt_T2_2,abs(Fold)<0.5)

T2_2_Sig <- combine(sig2foldT221,sig2foldT222)

rm(sig_wt_T2_2)

rm(sig2foldT121)

rm(sig2foldT122)

rm(sig2foldT221)

rm(sig2foldT222)

rm(sta3)

rm(sig3)

rm(test5)

rm(test6)

rm(test7)

rm(test8)

#repeat for the third time point

test10= NULL

test12= NULL

#WT vs TM1 and Tm2 at T4

for (j in seq(nrow(W4)))

{

ID_REF<-rownames(W4[j,]) #to extract the probe id

sta5<-t.test(W4[j,], T14[j,])$statistic #to run the t.test and extract the $statistic (i.e., the difference in the mean vale)

sig5<-t.test(W4[j,], T14[j,])$p.value #to (re)run the t.test and extract the significance value (i.e., p-value)

test9<-cbind(ID_REF,sta5,sig5) # this juxtaposing sta and sig

test10<-rbind(test10,test9) # to same sta and sig

ID_REF<-rownames(W4[j,]) #to extract the probe id

sta6<-t.test(W4[j,], T24[j,])$statistic #to run the t.test and extract the $statistic (i.e., the difference in the mean vale)

sig6<-t.test(W4[j,], T24[j,])$p.value #to (re)run the t.test and extract the significance value (i.e., p-value)

test11<-cbind(ID_REF,sta6,sig6) # this juxtaposing sta and sig

test12<-rbind(test12,test11) # to same sta and sig

}

test10<- as.data.frame(test10)

test10$Fold<- T14Fold1

#make a numeric dataset

test10$sta5<-as.numeric(as.character(test10$sta5))

test10$sig5<-as.numeric(as.character(test10$sig5))

sig_wt_T1_4 <-subset(test10,abs(sig5)<.05)

sig2foldT141 <-subset(sig_wt_T1_4,abs(Fold)>2)

sig2foldT142 <- subset(sig_wt_T1_4,abs(Fold)<0.5)

T1_4_Sig <- combine(sig2foldT141,sig2foldT142)

test12<- as.data.frame(test12)

test12$Fold<- T24Fold1

#make a numeric dataset

test12$sta6<-as.numeric(as.character(test12$sta6))

test12$sig6<-as.numeric(as.character(test12$sig6))

sig_wt_T2_4 <-subset(test12,abs(sig6)<.05)

sig2foldT241<-subset(sig_wt_T2_4,abs(Fold)>2)

sig2foldT242 <- subset(sig_wt_T2_4,abs(Fold)<0.5)

T2_4_Sig <- combine(sig2foldT241,sig2foldT242)

rm(sta4)

rm(sig4)

rm(sta5)

rm(sig5)

rm(sta6)

rm(sig6)

rm(sig_wt_T1_4)

rm(sig_wt_T2_4)

rm(sig2foldT141)

rm(sig2foldT142)

rm(sig2foldT241)

rm(sig2foldT242)

rm(sta3)

rm(sig3)

rm(test9)

rm(test10)

rm(test11)

rm(test12)

test14= NULL

test16= NULL

#WT vs TM1 and Tm2 at T6

for (j in seq(nrow(W6)))

{

ID_REF<-rownames(W6[j,]) #to extract the probe id

sta7<-t.test(W6[j,], T16[j,])$statistic #to run the t.test and extract the $statistic (i.e., the difference in the mean vale)

sig7<-t.test(W6[j,], T16[j,])$p.value #to (re)run the t.test and extract the significance value (i.e., p-value)

test13<-cbind(ID_REF,sta7,sig7) # this juxtaposing sta and sig

test14<-rbind(test14,test13) # to same sta and sig

ID_REF<-rownames(W6[j,]) #to extract the probe id

sta8<-t.test(W6[j,], T26[j,])$statistic #to run the t.test and extract the $statistic (i.e., the difference in the mean vale)

sig8<-t.test(W6[j,], T26[j,])$p.value #to (re)run the t.test and extract the significance value (i.e., p-value)

test15<-cbind(ID_REF,sta8,sig8) # this juxtaposing sta and sig

test16<-rbind(test16,test15) # to same sta and sig

}

test14<- as.data.frame(test14)

test14$Fold<- T16Fold1

#make a numeric dataset

test14$sta7<-as.numeric(as.character(test14$sta7))

test14$sig7<-as.numeric(as.character(test14$sig7))

sig_wt_T1_6 <-subset(test14,abs(sig7)<.05)

sig2foldT161 <-subset(sig_wt_T1_6,abs(Fold)>2)

sig2foldT162 <- subset(sig_wt_T1_6,abs(Fold)<0.5)

T1_6_Sig <- combine(sig2foldT161,sig2foldT162)

test16<- as.data.frame(test16)

test16$Fold<- T26Fold1

#make a numeric dataset

test16$sta8<-as.numeric(as.character(test16$sta8))

test16$sig8<-as.numeric(as.character(test16$sig8))

sig_wt_T2_6 <-subset(test16,abs(sig8)<.05)

sig2foldT261 <-subset(sig_wt_T2_6,abs(Fold)>2)

sig2foldT262 <- subset(sig_wt_T2_6,abs(Fold)<0.5)

T2_6_Sig <- combine(sig2foldT261,sig2foldT262)

rm(sta7)

rm(sig7)

rm(sta8)

rm(sig8)

rm(sig_wt_T1_6)

rm(sig_wt_T2_6)

rm(sig_wt_T1_2)

rm(sig2foldT161)

rm(sig2foldT162)

rm(sig2foldT261)

rm(sig2foldT262)

rm(test13)

rm(test14)

rm(test15)

rm(test16)

#Overlap the Datasets (Tm1 and Tm2)

T0DEGs <- merge(T1_0_Sig, T2_0_Sig, by = "ID_REF")

T2DEGs <- merge(T1_2_Sig, T2_2_Sig, by = "ID_REF")

T4DEGs <- merge(T1_4_Sig, T2_4_Sig, by = "ID_REF")

T6DEGs <- merge(T1_6_Sig, T2_6_Sig, by = "ID_REF")

#Put all DEGs AT numbers onto single files

T0AT <-as.data.frame(T0DEGs$ID_REF)

T2AT <-as.data.frame(T2DEGs$ID_REF)

T4AT <-as.data.frame(T4DEGs$ID_REF)

T6AT <-as.data.frame(T6DEGs$ID_REF)

#Remove all of the extra files!

rm(T10Fold1)

rm(T20Fold1)

rm(T12Fold1)

rm(T22Fold1)

rm(T14Fold1)

rm(T16Fold1)

rm(T24Fold1)

rm(T26Fold1)

rm(W0)

rm(W2)

rm(W4)

rm(W6)

rm(T10)

rm(T12)

rm(T14)

rm(T16)

rm(T20)

rm(T22)

rm(T24)

rm(T26)

rm(W0mean)

rm(W2mean)

rm(W4mean)

rm(W6mean)

rm(T10mean)

rm(T12mean)

rm(T14mean)

rm(T16mean)

rm(T20mean)

rm(T22mean)

rm(T24mean)

rm(T26mean)

rm(T1_0_Sig)

rm(T2_0_Sig)

rm(T1_2_Sig)

rm(T2_2_Sig)

rm(T1_4_Sig)

rm(T2_4_Sig)

rm(T1_6_Sig)

rm(T2_6_Sig)

rm(mydata)

RESULTS:

GENIE3 CODE:

#############################################################

#GENIE3 for TM_RNA_Seq, Run this on entire FPKM matrix

#use the TFS in the BAG list as the regulatory genes for the ML

#I will use individual transcriptomes, not averaged reps, to make the TF number equal the sample size

#balancing the TF# and sample size# helps prevent over or under fitting

### This is the list of BAG genes

BAGs<-`200415_BAGs`

### This is an expression matrix of full transcriptomes from the TM_RNA-seq project

FPKM <-TM_RNA_SEQ_FPKM

### This will merge the FPKM with the BAGs, remove gene names and reassign them as row names

BAG_FPKM <- merge(BAGs,FPKM, by = "Gene")

exprMatr <- BAG_FPKM[c(2:37)]

row.names(exprMatr) <- BAG_FPKM$Gene

### This is a list of TFs in the BAG list

### it was generated by finding the overlap between the BAG list and the list of all Arabidopsis TFs

regulators = c("AT1G01720",

"AT1G61660",

"AT4G00870",

"AT2G42380",

"AT3G58120",

"AT1G74930",

"AT4G01720",

"AT3G05690",

"AT2G45660",

"AT5G60910",

"AT4G37750",

"AT1G19350",

"AT3G50260",

"AT4G23750",

"AT1G71130",

"AT5G25190",

"AT4G28140",

"AT2G46270",

"AT3G28920",

"AT1G07900",

"AT5G59780",

"AT5G39610",

"AT1G52890",

"AT3G15500",

"AT1G77450",

"AT3G04060",

"AT1G69490",

"AT4G38340",

"AT4G27410",

"AT5G15800",

"AT3G12250",

"AT5G07100",

"AT4G18170",

"AT3G01970",

"AT2G46400",

"AT5G49520",

"AT1G69310",

"AT3G56400",

"AT5G59340",

"AT5G04340")

###Prep and Run GENIE3

library(GENIE3)

set.seed(123) # For reproducibility of results

weights <- GENIE3(as.matrix(exprMatr), regulators = regulators, verbose=TRUE)

### Isolate the lsit of regulatory links

linkList <- getLinkList(weights)

dim(linkList)

write.csv(linkList, file = "200714_BAG_GENIE3_Output.csv")

####add back the reannotated network

GRN<- AUPR_0_64_Cutoff_TM_GENIE3

names(GRN)[1] <- "Gene"

####Overlap the AT numbers with the Gene aliases from TAIR

#Keep only the first annotation for each gene for each AT number

AT_Names <-gene_names %>% distinct(locus_name, .keep_all = TRUE)

names(AT_Names)[1] <- "Gene"

#merge into Source gene column

GRN1<-merge(AT_Names,GRN, by = "Gene", all = TRUE)

GRN2 <-GRN1[!is.na(GRN1$Score), ]

names(GRN2)[2] <- "Source"

names(GRN2)[1] <- "Source_AT"

names(GRN2)[4] <- "Gene"

#### merge the target with the AT names

GRN3<- merge(GRN2, AT_Names, by = "Gene", all = TRUE)

GRN4 <-GRN3[!is.na(GRN3$Score), ]

names(GRN4)[1] <- "Target_AT"

names(GRN4)[7] <- "Target"

namedGRN<-GRN4[c(1:3,5:7)]

write.csv(namedGRN, file = "200714_Named_Trimmed_network.csv")

### I exported the network and maually annotated the names for genes with "NA"

GRN5<-X200714_Named_Trimmed_network[c(1:6)]

###### The following file contains the time of differential expression for each gene in T2 by edgeR

Time <-`200412_DEGTime_edgeR_T2`

names(Time)[2] <- "Target_AT"

GRN6 <-merge(GRN5, Time, by = "Target_AT", all = TRUE)

GRN7 <-GRN6[!is.na(GRN6$Score), ]

write.csv(GRN7, file = "GRN7.csv")

######taking the trend data from T2 and making it into a column format

Trend <-X200714_Trend_T2_Data

Trend_Data<-melt(Trend, id.vars = "Gene")

###Merge this with the Target Data

names(GRN8)[2] <- "Gene"

GRN9<- merge(GRN8, Trend_Data, by = "Gene", all = TRUE)

GRN10 <-GRN9[!is.na(GRN9$Score), ]

####subset to remoce excess values

GRN10$Match <-as.numeric(GRN10$`Target Time` == GRN10$variable)

GRN11<- subset(GRN10, Match == 1)

write.csv(GRN11, file = "GRN11.csv")

### I exported GRN11 and removed the match column, then reordered the columns.

names(Ath_TF_list)[2] <- "Gene"

names(GRN12)[4] <- "Gene"

GRN13<-merge(Ath_TF_list,GRN12, by = "Gene", all = TRUE)

GRN14 <-GRN13[!is.na(GRN13$Score), ]

names(GRN14)[1] <- "Target_AT"

names(GRN14)[2] <- "Target_TFs"

names(GRN14)[6] <- "Gene"

GRN15<-merge(Ath_TF_list,GRN14, by = "Gene", all = TRUE)

GRN16 <-GRN15[!is.na(GRN15$Score), ]

write.csv(GRN16, file = "GRN16.csv")

##### reorganized the GRN16 file to annotate which are TFs

##forgot to add time for the source nodes, adding here

names(Time)[2] <- "Gene"

GRN18 <-merge(GRN17, Time, by = "Gene", all = TRUE)

GRN19 <-GRN18[!is.na(GRN18$Score), ]

write.csv(GRN19, file = "GRN19.csv")

###manuallly trimmed GRN19, importing back

names(GRN20)[5] <- "Gene"

GRN21<-merge(X200628_LSD, GRN20, by = "Gene", all = TRUE)

GRN22 <-GRN21[!is.na(GRN21$Score), ]

names(GRN22)[1] <- "Target_AT"

names(GRN22)[2] <- "Target_Role"

names(GRN22)[4] <- "Gene"

GRN23<-merge(X200628_LSD, GRN22, by = "Gene", all = TRUE)

GRN24 <-GRN23[!is.na(GRN23$Score), ]

names(GRN24)[1] <- "Source_AT"

names(GRN24)[2] <- "Source_Role"

write.csv(GRN24, file = "GRN24.csv")

#### GRN25 will be overlapped with DILS data for label colors in cytoscape

names(GRN25)[6] <- "Gene"

GRN26<- merge(GRN25,`200424DILS_DEGs_log2`, by = "Gene", all = TRUE)

GRN27 <-GRN26[!is.na(GRN26$Score), ]

names(GRN27)[1] <- "Target_AT"

names(GRN27)[15] <- "Target_DILS"

names(GRN27)[2] <- "Gene"

GRN28<- merge(GRN27,`200424DILS_DEGs_log2`, by = "Gene", all = TRUE)

names(GRN28)[1] <- "Source_AT"

GRN29 <-GRN28[!is.na(GRN28$Score), ]

names(GRN29)[21] <- "Source_DILS"

write.csv(GRN29, file = "GRN29.csv")

PC3 Separation: We include this to show that at each time point, there is slight movement across PC3

.

###### NEW PCA ANALYSIS

mydata<-round(FPKMafterfilter[c(2:37)])

row.names(mydata) <- FPKMafterfilter$z

library(DESeq2)

dds_TM <- DESeqDataSetFromMatrix(countData = mydata,

colData = X200911_TM_PCA_Metadata ,

design = ~ Genotype + Time)

library(ggplot2)

vsd_root <- vst(dds_TM, blind=TRUE)

pcaDataroot <- plotPCA(vsd_root, intgroup=c("Genotype", "Time"), returnData = TRUE)

percentVarroot <-round(100 * attr(pcaDataroot, "percentVar"))

sp <- ggplot(pcaDataroot, aes(PC4, PC5, color= Genotype, shape=Time)) +

geom_point(size=3) +

xlab(paste0("PC4: ",percentVarroot[4],"% variance")) +

ylab(paste0("PC5: ",percentVarroot[5],"% variance")) +

coord_fixed()

sp

#####################################################################################

#################################################################################### After filtering, transcriptome had 19438 genes, so this covers entire transcriptome

plotPCA <-function (object, intgroup = "condition", ntop = 20000, returnData = FALSE)

{

rv <- rowVars(assay(object))

select <- order(rv, decreasing = TRUE)[seq_len(min(ntop,

length(rv)))]

pca <- prcomp(t(assay(object)[select, ]))

percentVar <- pca$sdev^2/sum(pca$sdev^2)

if (!all(intgroup %in% names(colData(object)))) {

stop("the argument 'intgroup' should specify columns of colData(dds)")

}

intgroup.df <- as.data.frame(colData(object)[, intgroup, drop = FALSE])

group <- if (length(intgroup) > 1) {

factor(apply(intgroup.df, 1, paste, collapse = " : "))

}

else {

colData(object)[[intgroup]]

}

d <- data.frame(PC4 = pca$x[, 4], PC5 = pca$x[, 5], group = group,

intgroup.df, name = colData(object)[,1])

if (returnData) {

attr(d, "percentVar") <- percentVar[1:5]

return(d)

}

ggplot(data = d, aes_string(x = "PC1", y = "PC2", color = "group", label = "name")) + geom_point(size = 3) + xlab(paste0("PC4: ", round(percentVar[4] * 100), "% variance")) + ylab(paste0("PC5: ", round(percentVar[5] * 100), "% variance")) + coord_fixed() + geom_text_repel(size=3)

}

###########################################

##########################################
